# Metronidazole-triazole conjugates: Activity against *Clostridium difficile* and parasites

**DOI:** 10.1016/j.ejmech.2015.06.019

**Published:** 2015-08-28

**Authors:** Angie M. Jarrad, Tomislav Karoli, Anjan Debnath, Chin Yen Tay, Johnny X. Huang, Geraldine Kaeslin, Alysha G. Elliott, Yukiko Miyamoto, Soumya Ramu, Angela M. Kavanagh, Johannes Zuegg, Lars Eckmann, Mark A.T. Blaskovich, Matthew A. Cooper

**Affiliations:** a Division of Chemistry and Structural Biology, Institute for Molecular Bioscience, The University of Queensland, Brisbane, Queensland, 4072, Australia; b Skaggs School of Pharmacy and Pharmaceutical Sciences, University of California, San Diego, La Jolla, CA, 92093, USA; c Department of Medicine, University of California, San Diego, La Jolla, CA, 92093, USA; d Marshall Centre for Infectious Diseases Research and Training, University of Western Australia, Perth, Western Australia, 6099, Australia

**Keywords:** Nitroimidazole, Click chemistry, Antibiotic, Anaerobe, MIC, minimum inhibition concentration, Mtz, metronidazole, MtzS, metronidazole sensitive, MtzR, metronidazole resistant

## Abstract

Metronidazole has been used clinically for over 50 years as an antiparasitic and broad-spectrum antibacterial agent effective against anaerobic bacteria. However resistance to metronidazole in parasites and bacteria has been reported, and improved second-generation metronidazole analogues are needed. The copper catalysed Huigsen azide-alkyne 1,3-dipolar cycloaddition offers a way to efficiently assemble new libraries of metronidazole analogues. Several new metronidazole-triazole conjugates (Mtz-triazoles) have been identified with excellent broad spectrum antimicrobial and antiparasitic activity targeting *Clostridium difficile*, *Entamoeba histolytica* and *Giardia lamblia*. Cross resistance to metronidazole was observed against stable metronidazole resistant *C. difficile* and *G. lamblia* strains. However for the most potent Mtz-triazoles, the activity remained in a therapeutically relevant window.

## Highlights

• A series of 20 metronidazole (Mtz)-triazole conjugates were synthesised.
• Several Mtz-triazoles had increased potency against pathogenic anaerobes.
• The compounds were not cytotoxic at 100 μM against mammalian cell lines.
• Cross-resistance was observed against Mtz-resistant bacteria and parasites.
• Therapeutically relevant activity was retained against Mtz-resistant *G. lamblia*.

## Graphical abstract

**Figure:**
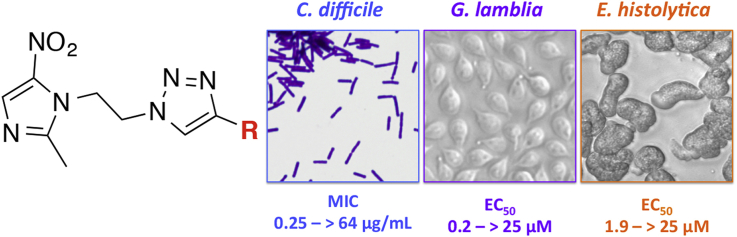

## 1 Introduction

For over 50 years the 5-nitroimidazole antibiotic metronidazole **1** has been in clinical use as a broad-spectrum agent for treatment of Gram-positive and Gram-negative anaerobic bacterial infections as well as parasitic infections [1]. Reduction of the nitro group to the nitro radical anion by electron carriers in an anaerobic environment leads to decomposition to form toxic metabolites, which cause DNA damage and nonspecific macromolecular damage leading to cell death [2]. However, metronidazole resistance has been observed in both parasites [3,4] and anaerobic bacteria [5,6]. Over the last ten years metronidazole has also been extensively used to treat *Clostridium difficile* infection (CDI), an intestinal infection that causes life-threatening severe diarrhea, abdominal pain and fever [7]. The spores produced by *C. difficile* are resistant to heat and alcohol based disinfectants, complicating eradication and promoting hospital-based outbreaks. Metronidazole is one of three antibiotics routinely prescribed to treat the disease [8]. Metronidazole is effective against mild to moderate *C. difficile* infection, but, while not wide-spread, resistance has been observed in clinical isolates [9]. To this end, the development of next generation metronidazole analogues that can overcome resistance is therapeutically important.

**Fig. 1:**
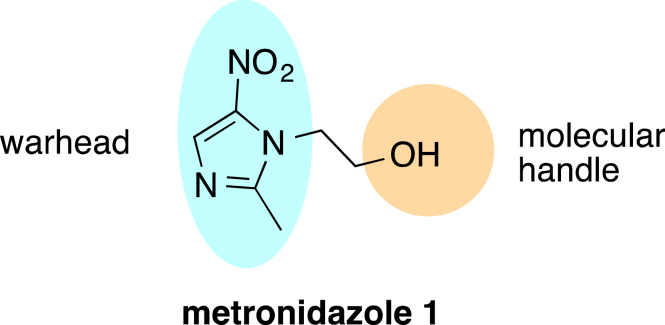
Metronidazole contains the 5-nitroimidazole warhead and a hydroxyl group amenable to modification.

Beena et al. previously reported the synthesis of metronidazole-triazole conjugates (Mtz-triazoles) with potent activity (reported as IC_50_ values 0.06–0.35 μg/mL) against the facultative anaerobic bacteria *Staphylococcus aureus*, S*taphylococcus epidermis*, *Escherichia coli* and *Pseudomonas aeruginosa* [10]. Metronidazole is generally not active against Gram-negative and Gram-positive facultative anaerobic bacteria, so the potent activities of Mtz-triazoles observed by Beena et al. are suggestive of a different mode of action to metronidazole. Given that the active 5-nitroimidazole core (“warhead”) in metronidazole is maintained in Mtz-triazoles, and that the hydroxyl group of metronidazole is amenable to modification [11] (Fig. 1), we reasoned that this class might also exhibit potent activity against the anaerobic bacteria and parasites for which metronidazole is used as a treatment. Therefore, we believed Mtz-triazoles warranted further investigation for both antimicrobial and antiparasitic activity under anaerobic conditions.

Independently, during the course of this work, Miyamoto et al. [12] reported a similar approach to Mtz-triazoles, prepared by reaction of six different 5-nitroimidazole azides with a library of alkynes. The compounds were prepared for testing by dilution of the crude reaction mixtures (>85% purity by LCMS) with dimethyl sulfoxide. This methodology bypassed the bottleneck of compound purification and characterization and allowed for the rapid assessment of activity. The solutions of crude material were tested against the parasites *Giardia lamblia* and *Trichomonas vaginalis*, the microaerophilic bacteria *Helicobacter pylori*, the anaerobes *C. difficile* and *Bacteroides fragilis* and the facultative anaerobic bacteria *E. coli*. It is possible that the crude mixtures contained unreacted starting azide **3**, which we have shown in this study retains activity, and may influence the reported crude compound potency. A key finding by Miyamoto was that Mtz-triazoles were often active against metronidazole resistant (MtzR) strains of *T. vaginalis* (47% of compounds) and *G. lamblia* (100% of compounds), but not against *H. pylori frxA* and *rdxA* double mutant (1.4% of compounds) [12]. From this result, we hypothesized that Mtz-triazoles may possess activity against metronidazole resistant *C. difficile*. More recently Beena et al. described the activity of Mtz-triazoles against the anaerobic protozoan *Entamoeba histolytica* [13]. They tested a library of 10 Mtz-triazoles and found **4a**, **4h**, **4p** and **4q** to have potent activity (IC_50_ = 0.008–2.36 μM) against *E. histolytica* HM1: IMSS [13].

**Scheme 1:**
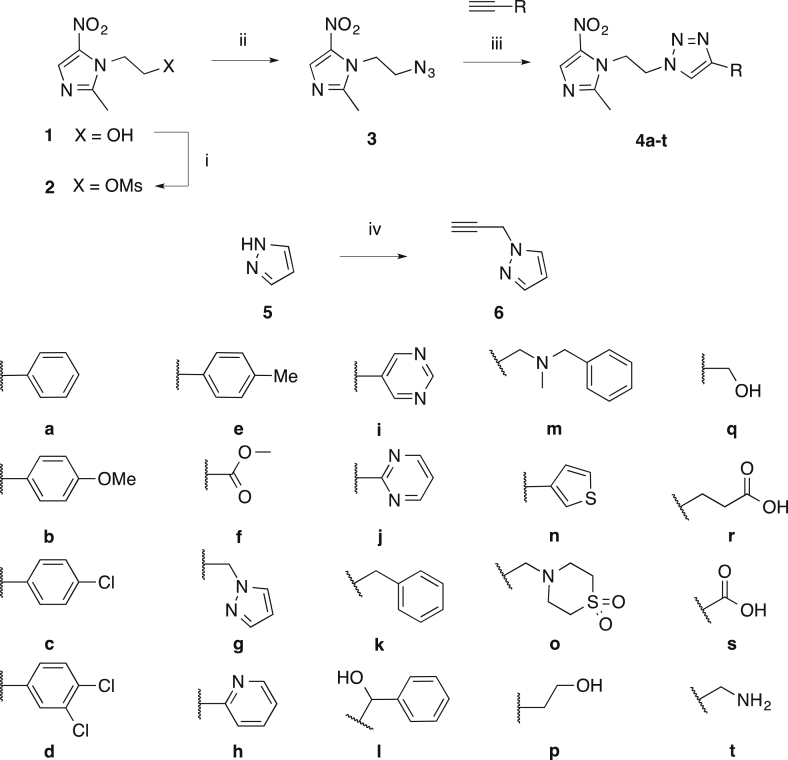
Synthesis of metronidazole-triazole conjugate library. i) MsCl, TEA, DCM, 2 °C to rt, 80 min, 85% yield ii) NaN_3_, DMF, 50 °C, 21 h, quant. yield iii) CuSO_4_, sodium ascorbate, MeOH, rt or 45 °C, 20 min–48 h, 9–97% iv) propargyl bromide (80% w/v in toluene), K_2_CO_3_, TBAB, rt, 3 days, 16%.

We now report an expansion and advancement of these approaches with the synthesis of a small library of purified Mtz-triazoles **4a-t** (>95% purity, Scheme 1), including ten novel derivatives (**4b**, **4c**, **4f, 4g**, **4i**, **4j**, **4l**, **4o**, **4s** and **4t**). We also explore the antimicrobial and antiparasitic properties of the set of Mtz-triazoles and the parent azide **3** (>95% purity) and determine their activity against additional microbial targets that have not previously been examined. We evaluated the activity of Mtz-triazoles against the parasites *E. histolytica* and *G. lamblia*, multiple pathogenic strains of the anaerobe *C. difficile*, the microaerophile *H. pylori* and the facultative anaerobic bacterial ESKAPE pathogens *E. coli*, methicillin resistant *S. aureus* (MRSA), *Klebsiella pneumoniae*, *Acinetobacter baumanni* and *P. aeruginosa*. The antibacterial activity was measured against a stable MtzR *C. difficile* strain (CD26A54_R), the parent *C. difficile* strain with elevated metronidazole minimum inhibitory concentration (MIC) (CD26A54_S) and MtzR *H. pylori* clinical isolates, while cytotoxicity of the compounds was evaluated against mammalian liver (HepG2) and kidney (HEK293) cell lines.

## 2 Materials and methods

### 2.1 Synthesis of Mtz-triazole library

Mtz-triazoles were synthesized from metronidazole via an azide intermediate by activation of the hydroxyl group with methanesulfonyl chloride (Scheme 1). Displacement of methanesulfonate **2** with sodium azide provided the desired azide **3** by nucleophilic substitution. The azide substituent was then reacted with a library of alkynes to give **4a-t** by copper-catalysed Huisgen 1,3-dipolar cycloaddition with copper sulfate and sodium ascorbate in methanol, with heating to 45 °C and/or additional reagents added if monitoring indicated the reaction was incomplete. All alkynes utilized were commercially available with the exception of pyrazole alkyne **6**. Pyrazole alkyne **6** was prepared by reacting pyrazole **5** with propargyl bromide in the presence of potassium carbonate and *t*-butylammonium bromide as a phase transfer catalyst (Scheme 1) [14]. All compounds were characterized by ^1^H and ^13^C NMR, LCMS and HRMS and detailed experimental procedures and characterization are provided in the supplementary information.

### 2.2 MIC assays

All compounds were tested for activity against a set of anaerobic *C. difficile* bacteria (630 ATCC BAA-1382, NAP1/027 M7404, NAP1/027 ATCC BAA-1803, VPI10463 ATCC 43255, CD26A54_S and CD26A54_R) and representative ESKAPE pathogens *S. aureus* (MRSA ATCC 43300), *E. coli* (ATCC 25922), *K. pneumoniae* (ATCC 700603), *A. baumannii* (ATCC 19606) and *P. aeruginosa* (ATCC 27853) using a standard broth microdilution assay. Selected compounds were tested for activity against *H. pylori* strains (26695, and clinical isolates MtzS 13/25, MtzR 98/285 and MtzR 13/61) using agar plate dilution, and for antimicrobial activity against twelve Gram-positive bacterial strains: *Enterococcus faecalis* (VanA clinical isolate), *Enterococcus faecium* (MDR VanA ATCC 51559), *Streptococcus pneumoniae* (MDR ATCC 700677), *S. aureus* (MRSA ATCC 43300, MRSA clinical isolate, MRSA/DRSA clinical isolate, GISA NARSA NRS 1, GISA NARSA NRS 17, VRSA NARSA VRS1, VRSA NARSA VRS4 and VRSA NARSA VRS10) using broth microdilution. *C. difficile* strains were grown at 37 °C in a COY anaerobic chamber (5% H_2_, 10% CO_2_, 85% N_2_). *H. pylori* strains were grown at 37 °C under a microaerobic atmosphere (5% O_2_, 10% CO_2_). All facultative anaerobic bacteria were grown at 37 °C with normal atmospheric oxygen levels. All experiments were performed in duplicate with metronidazole, vancomycin, linezolid or colistin as positive controls for relevant strains (see Table 1). Positive growth control of bacteria and DMSO as well as a negative control of only media were included for every plate. Full assay details are provided in the supplementary information.

### 2.3 Antiparasitic assays

Compounds were tested for antiparasitic activity against *E. histolytica* (HM1:1MSS strain) and *G. lamblia* (WB, BRIS/87/HEPU/713 (713) [15], BRIS/83/HEPU/106 (106) [16] and the metronidazole resistant syngeneic line 713-M3 [15,16]) using an ATP-bioluminescence based screen for cell growth and survival [17,18]. Assay plates were inoculated with trophozoites (5 × 10^3^ parasites/well) and incubated in the GasPak™ EZ Anaerobe Gas Generating Pouch Systems (VWR, West Chester, PA) to maintain anaerobic conditions throughout the incubation period. The assays were performed in triplicate using the CellTiter-Glo Luminescent Cell Viability Assay [17]. Metronidazole was used as a positive control.

### 2.4 Cytotoxicity

Compounds were tested for cytotoxicity against mammalian HepG2 and HEK293 cell lines, as detailed in the supplementary information.

## 3 Results and discussion

### 3.1 Design of metronidazole-triazole conjugate library

The Mtz-triazole library was designed to contain a variety of structural groups with some compounds identical to those reported by Beena et al. (**4a**, **4h**, **4p** and **4q**) [10]. Initial results against *C. difficile* showed that hydrophobic **4a** (R = phenyl) maintained activity relative to metronidazole **1** while more hydrophilic **4p** (R = hydroxyethyl) and **4q** (R = hydroxymethyl) lost activity. Therefore the subsequent library was biased towards exploring variance of the aromatic ring substitutions, extension of the position of the aromatic ring relative to the triazole core and replacement of the phenyl group with various heterocycles, with several of the latter selected due to their rating in terms of medicinal chemistry ‘developability’ [19]. Amine **4t**, and acids **4r** and **4s** were included to explore the structure activity relationships of non-aromatic ionisable groups. Compounds **4a**, **4d**, **4e**, **4h**, **4k**, **4m**, **4n**, **4q** and **4r** were reported by Miyamoto [12], but **4a**, **4h** and **4q** were not tested against *C. difficile*. Compounds **4b**, **4c**, **4f, 4g**, **4i**, **4j**, **4l**, **4o**, **4s** and **4t** are described for the first time.

### 3.2 Antimicrobial and antiparasitic activity of metronidazole-triazole conjugates

The antimicrobial and antiparasitic spectrum of action of Mtz-triazoles was assessed against the anaerobic bacteria *C. difficile* and the anaerobic parasites *E. histolytica* and *G. lamblia* (Table 1). Variations to the phenyl group of **4a** were well tolerated and activity of **4b-e** was maintained against *C. difficile*, *E. histolytica* and *G. lamblia* (Table 1). Phenyl derivatives **4a-e** were several fold more active against *G. lamblia* than metronidazole. Pyridine **4h** and thiophene **4n** heterocycles also possessed broad-spectrum activity against *C. difficile*, *E. histolytica* and *G. lamblia*. However, we found that **4h** was not as potent compared to metronidazole in the ATP-bioluminescence parasite assay in contrast to the eosin-stain method used by Beena et al. [13] The pyrazole **4g** and pyrimidine **4i-j** maintained activity against *C. difficile* within one to two 2-fold dilutions of metronidazole. Compounds **4g** and **4i-j** were still active against *G. lamblia* compared to metronidazole, but were several fold less potent than the phenyl derivatives **4a-e**. The pyrazole **4g** and pyrimidines **4i**-**j** did not inhibit *E. histolytica* at 25 μM, showing that the R group can be used as a handle to tune the selectivity of this class towards different organisms. Compound **4k** (benzyl) and **4m** (CH_2_NMe-benzyl) maintained broad-spectrum activity but **4l** (CHOH-phenyl) was inactive against *E. histolytica* at 25 μM.

Several compounds were inactive, or weakly active, against all three microorganisms at the highest concentration tested. These included the non-aromatic thiomorpholine dioxide **4o**, amine **4t** and the carboxylic acids **4r** and **4s**. The methyl hydroxyl **4q** and ethyl hydroxyl **4p** were inactive against *E. histolytica* and *G. lamblia* and weakly potent against *C. difficile*, continuing the trend of reduced activity with more polar substituents.

We also demonstrate that the parent azide **3** possesses potent activity against *C. difficile*, *E. histolytica* and *G. lamblia*. Therefore, when assessing combinatorial-like libraries of crude material for biological activity, the activity of any unreacted parent compounds is an important consideration, particularly when the compound warhead is maintained.

The MICs of **1**, **3** and **4a-t** were >32 μg/mL against the representative ESKAPE pathogens (MRSA, *E. coli*, *K. pneumoniae*, *A. baumannii* and *P. aeruginosa*) tested under aerobic conditions. In addition, the MIC values of a subset of compounds (metronidazole **1**, azide **3, 4a** (phenyl) and **4q** (CH_2_OH)) were all >64 μg/mL against a panel of 8 additional drug resistant *S. aureus* strains, vancomycin resistant *E. faecalis*, vancomycin resistant *E. faecium* and multidrug resistant *S. pneumoniae*. This lack of activity is contrary to Beena's report, but consistent with the reported inactivity of 378 Mtz-triazoles against *E. coli* in Miyamoto's study, and the inactivity of metronidazole against facultative anaerobic bacteria.

### 3.3 Activity of metronidazole-triazole conjugates against C. difficile strain panel

The activity of the Mtz-triazoles did not vary significantly against multiple strains of *C. difficile*, including two major pathogenic strains of NAP1/027 and a VPI10463 strain associated with epidemics (Table 1). The MICs of individual compounds typically remained within one 2-fold dilution against the four strains of *C. difficile* tested. This is important, as viable drug candidates must possess appropriate broad strain coverage. A review of antibiotic drug candidates in development found that activity against multiple strains of *C. difficile* could vary substantially [20]. This can be explained since *C. difficile* has a highly mobile, mosaic genome and there is wide strain diversity between isolates [21,22]. Isolates can be divided across five main phylogenetic clades, multiple ribotypes and toxinotypes [23]. Recently, genomic epidemiology studies examining and tracking *C. difficile* outbreaks in Europe found that a high proportion of strains causing infection were not related to prior infectious strains [22]. Therefore, there is a large pool of genetically diverse strains in the community and selection and spread of intrinsically resistant strains could occur. However, the broad-strain activity of Mtz-triazoles against *C. difficile* in this study encourages further development of this class.

### 3.4 Activity of metronidazole-triazole conjugates against metronidazole resistant C. difficile

While metronidazole resistance has been reported in the clinic [9,24], performing antimicrobial susceptibility testing against resistant *C. difficile* isolates is problematic since the resistance phenotype is unstable and often reported to be lost on freeze thaw cycles or on passaging of the isolates [5]. However, Lynch et al. have reported a stable metronidazole resistant (MtzR) *C. difficile* clone [25]. The activity of Mtz-triazoles was assessed against this stable MtzR strain of *C. difficile* (CD26A54_R) and the parent metronidazole sensitive (MtzS) (CD26A54_S) strain, which lost the metronidazole resistance on freeze thawing, but still retained a slightly elevated MIC value to metronidazole compared to the control MtzS NAP1/027 strain. The MIC to metronidazole in BHIS broth at 48 h was lower than the metronidazole MIC determined by E-test on BAKHS (4 μg/mL vs 48 μg/mL), similar to values reported by Chong et al. (8 μg/mL vs > 32 μg/mL) [26].

The resistance to metronidazole became more apparent on incubation in broth for 48 h (MIC = 2 μg/mL after 24 h incubation vs MIC = 4 μg/mL after 48 h) (Supplementary Table 1). In contrast, MICs against the control MtzS NAP1/027 strain ATCC 1803 did not change significantly between 24 h and 48 h (Supplementary Table 1). Although several compounds (**4a**, **4e** and **4n**) were active against MtzR *C. difficile* at 1 dilution lower than metronidazole (MIC = 2 μg/mL vs 4 μg/mL), none of the Mtz-triazoles were active against MtzR *C. difficile* at levels comparable to the activity of metronidazole against MtzS strains (MIC = 0.5 μg/mL). Reduced activity against the parent *C. difficile* strain CD26A54_S with intermediary metronidazole susceptibility was also observed.

### 3.5 Activity of metronidazole-triazole conjugates against additional G. lamblia strains including metronidazole resistant G. lamblia

Selected compounds (**4a-f**, **4h**, **4k-n**) were tested against two additional strains of MtzS *G. lamblia* (106 and 713) and one MtzR strain (713-M3) derived from the parent MtzS strain 713 (Table 2). The Mtz-triazoles displayed similar activity against the additional MtzS strains (106 and 713) compared to *G. lamblia* strain WB. Encouragingly, the Mtz-triazoles were all more potent than metronidazole against MtzR *G. lamblia* 713-M3. However, with the exception of **4d**, all of the Mtz-triazoles lost activity against the MtzR *G. lamblia* 713-M3 when compared to the parent MtzS strain 713, similar to the loss of activity against MtzR *C. difficile*. Mtz-triazoles of scaffold **4** were also all observed by Miyamoto et al. to lose activity against MtzR *G. lamblia* 713-M3 and 106-2ID10. However, while activity diminished against the MtzR 713M strain, the Mtz-triazoles were often still much more potent than metronidazole itself, meaning that the EC_50_ remained within a therapeutically meaningful window.

The activities of purified Mtz-triazoles (>95% purity) against *G. lamblia* strains 106, 713 and 713M were compared with the literature activities [12] determined with crude reaction mixtures (Supplementary Table 3). The activities were generally in close agreement, supporting the methodology used by Miyamoto [12], although a 3.7–8 fold difference was observed for Mtz-triazoles **4d**, **4e** and **4m** against 1 strain of *G. lamblia*.

### 3.6 Activity of metronidazole-triazole conjugates against metronidazole resistant H. pylori

Selected compounds (**4f-h**, **4m** and **4s**) were tested against a panel of *H. pylori* strains using the CLSI agar dilution susceptibility method (Table 3) [27]. These strains included the reference strain 26695 and three clinical isolates 13/25 (metronidazole E-test MIC = 2 μg/mL), 98/285 (E-test MIC = 24 μg/mL) and 13/61 (E-test MIC = 256 μg/mL) with a range of resistance levels to metronidazole determined by the E-test. The susceptibilities of the strains to metronidazole determined by the agar dilution method differed from the susceptibilities obtained from the E-test as has been described previously [28]. The methyl ester **4f** was weakly active against *H. pylori*, while the carboxylic acid **4s** was inactive against all *H. pylori* strains as observed with *E. histolytica* and *G. lamblia* as well. Compounds **4g**, **4h** and **4m** were more active than metronidazole against the MtzS strains (26695 and clinical isolate 13/25) and 1–2 dilutions more active than metronidazole against the MtzR strains (clinical isolates 98/285 and 13/61) but they could not completely overcome metronidazole resistance, similar to the loss of activity against metronidazole resistant *C. difficile*.

### 3.7 Cytotoxicity

All compounds, including metronidazole **1**, azide **3**, and **4a-t** showed no cytotoxicity against HepG2 and HEK293 cells at concentrations up to 100 μM (CC_50_). This is consistent with the findings by Miyamoto et al. which did not observe cytotoxicity against HeLa cells at 50 μM [12].

### 3.8 Relationship of compound activity with compound properties

**Fig. 2:**
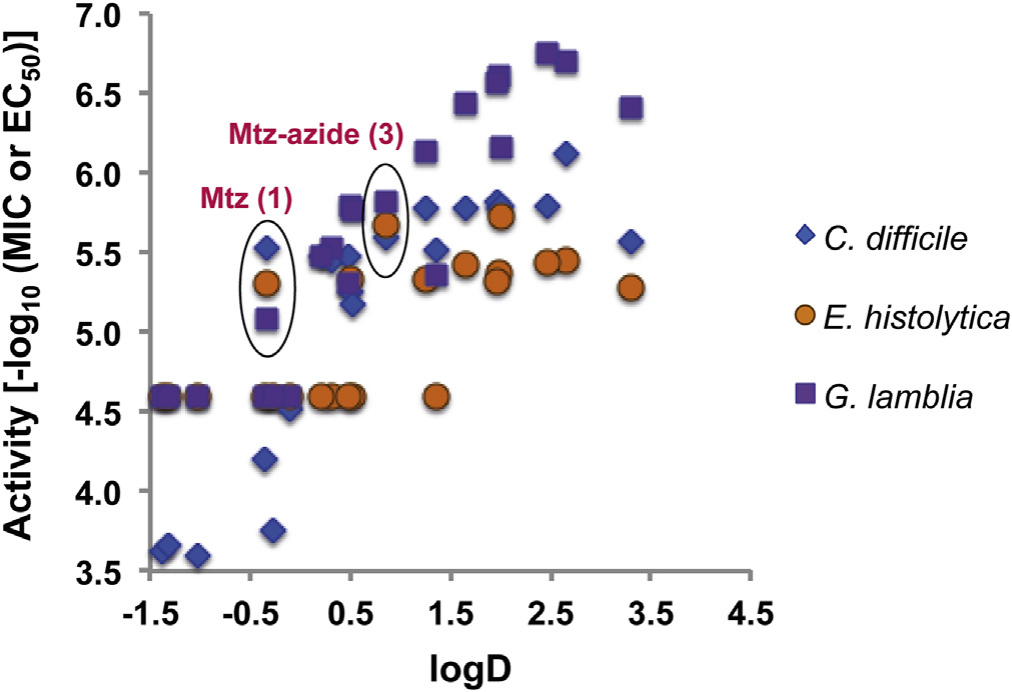
Correlation of activity against *G. lamblia*, *E. histolytica* and *C. difficile* with logD. Compounds with activity against parasites (EC_50_ < 25 μM) and *C . difficile* (MIC ≤ 8 μg/mL) have pEC50 or pMIC > 5.0.

In order to understand the relationship between compound properties and activity, compound properties including logP (or logD at pH = 7.4), molecular weight, topological polar surface area (tPSA), and predicted solubility (logS) were examined for correlation with activity against MtzS strains of *C. difficile*, *E. histolytica* and *G. lamblia*, with the activity expressed as pMIC (-log_10_MIC) or pEC_50_ (-log_10_EC_50_)) (Supplementary Table 4). Inactive compounds were included in the analysis at the highest concentration of compound tested. A correlation can be observed between the logD values and the activity against *G. lamblia* (with R^2^ (linear regression) = 0.84, Supplementary Fig. 1) (Fig. 2). The logD of the most potent compounds (**4a-e**, **4h**, **4k** and **4n**) against *G. lamblia* is between 1.5 and 3.0, with activity decreasing with lower logD. In contrast, only a mild correlation could be detected between logD and the activity against *C. difficile* (R^2^ = 0.68, Supplementary Fig. 1) and no correlation for activity against *E. histolytica* (R^2^ = 0.48, Supplementary Fig. 1). This might explain why the compounds with aromatic phenyl, thiophene and pyridine groups were more potent against *G. lamblia* than *E. histolytica* and *C. difficile*. Increasing the hydrophobicity (logD) of compounds to improve potency is generally considered undesirable in drug development due to the tendency for increased metabolism [29] and increased promiscuity [30]. However, in this case the antibiotic metronidazole has a low logD to begin with, such that the relatively higher logD of the compounds explored in this study does not place the compounds in an undesirable chemical space. On the other hand, the site of infection of *G. lamblia* is the small intestine, where low (<1) or high (>3) logD values [29], associated with reduced systemic uptake, might be beneficial for efficacy. However, metronidazole is 100% oral bioavailable [31] and so the importance of bioavailability for *in vivo* efficacy against *G. lamblia* infections is unclear.

## 4 Conclusion

Mtz-triazoles were synthesized and assessed for activity against the anaerobe *C. difficile*, microaerophile *H. pylori*, the parasites *E. histolytica* and *G. lamblia* as well as facultative anaerobic Gram-positive and Gram-negative bacteria. While no activity was observed against the facultative anaerobic bacteria, nine compounds were identified with potent broad spectrum activity against anaerobic organisms, while having no cytotoxicity against mammalian cell lines. Hydrophobic R groups such as the benzyl **4k**, varyingly substituted phenyl derivatives **4a-e** and heterocyclic R groups such as pyridine **4h** and thiophene **4n** were well tolerated and favored broad spectrum anaerobic activity. In contrast, polar R groups including pyrimidines **4i-j**, carboxylic acids **4p** and **4r** and methyl amine **4t** either resulted in loss of broad spectrum activity or were inactive against all organisms tested.

While active Mtz-triazoles displayed a narrow MIC range against multiple strains of *C. difficile* and *G. lamblia*, there was metronidazole cross resistance against the stable MtzR *C. difficile* strain CD26A54_R, MtzR *G. lamblia* strain 713M and clinical isolates of MtzR *H. pylori*. Mtz-triazoles **4a-t** generally lost activity against MtzR *C. difficile*, MtzR *G. lamblia* and MtzR *H. pylori.* However, since the Mtz-triazoles were often much more potent than metronidazole against *G. lamblia*, the EC_50_ remained within a therapeutically meaningful window. It is possible that surveying a more diverse chemical space of R group substituents or using alternative 5-nitroimidazole scaffolds could provide access to Mtz-triazoles that are even more potent and therefore active against MtzR strains of *C. difficile* and *G. lamblia*. Future work will focus on identifying such compounds and explore their *in vivo* efficacy against anaerobic pathogens.

## Author contribution

Planned experiments: AMJ, TK, AD, LE, MAB, MAC.

Performed experiments: AMJ, AD, CYT, JH, GK, AG, YM, SR, AK, JZ.

Wrote paper: AMJ, TK, LE, MAB, MAC.

## Conflict of interest

The authors declare no competing financial interest.

## Figures and Tables

**Table 1:** Activity of Mtz-triazoles against *C. difficile*, ESKAPE bacteria, *E. histolytica* and *G. lamblia*.

Compound		MIC (μg/mL)							EC_50_*(μM)*	
		*C. difficile*^a^						*ESKAPE bacteria*^*h*^	*E. histolytica*	*G. lamblia*
		630^*b*^	VPI 10463^*e*^	NAP1/027^*c*^	NAP1/027^*d*^	NAP1/027^f^	NAP1/027^g^		HM1:IMSS	WB
		MtzS	MtzS	MtzS	MtzS	MtzS	MtzR		MtzS	MtzS
**1**	metronidazole	0.5	0.5	0.5	0.5	1	4	>32	5	7.9
**3**	azide	1	0.5	0.5	0.5	2	8	>32	2.1	1.5
**4a**	phenyl	0.5	0.25-0.5	0.5	0.5	1	2	>32	4.2	0.25
**4b**	p-OMe-phenyl	0.5	0.25-0.5	0.5	0.5	1	4	>32	4.8	0.27
**4c**	p-Cl-phenyl	0.5	0.25	0.25	0.25	2	16	>32	3.6	0.20
**4d**	p,m-Cl_2_-phenyl	0.5	0.5-1	1	1	1	8-16	>32	5.2	0.39
**4e**	p-Me-phenyl	0.5	0.25	0.5	0.5	1	2	>32	3.7	0.18
**4f**	methyl ester	1	1	1	1	2	4	>32	>25	3.0
**4g**	pyrazole	1	1	1	1	2	4	>32	>25	4.9
**4h**	pyridine	0.5	0.5	0.5	0.5	1	4	>32	4.6	0.74
**4i**	5-pyrimidine	1	1	0.5-1	1	1	4	>32	>25	3.3
**4j**	2-pyrimidine	2	2	2	2	2-4	4	>32	>25	1.7
**4k**	benzyl	0.5	0.25-0.5	0.5	0.5	1	4	>32	1.9	0.70
**4l**	CHOH-phenyl	1	0.5	1	0.5-1	2	4	>32	>25	4.4
**4m**	CH_2_NMe-benzyl	2	1-2	1	2	2	8	>32	4.6	1.6
**4n**	thiophene	0.5	0.25-0.5	0.5	0.5	1	2	>32	3.8	0.37
**4o**	thiomorpholine	>64	>64	>64	>64	>64	>64	>32	>25	>25
**4p**	CH_2_CH_2_OH	8	8	16	8	8	16	>32	>25	>25
**4q**	CH_2_OH	16	16	16-32	16	16-32	16	>32	>25	>25
**4r**	CH_2_CH_2_COOH	>64	>64	>64	>64	>64	>64	>32	>25	>25
**4s**	COOH	64	64	64	64	>64	>64	>32	>25	>25
**4t**	CH_2_NH_2_	>64	>64	>64	>64	>64	>64	>32	>25	>25

a MIC results determined against CD26A54_S and CD26A54_R after 48 h growth, all other *C. difficile* strain MIC results determined at 24 h. See Supplementary Table 1 for MIC at 24 h for CD26A54_S and CD26A54_R and at 48 h for ATCC BAA-1803. *C. difficile* MICs are the median of at least n = 4, except for CD26A54_R where n = 8. ESKAPE pathogen MICs were performed in a single concentration screen with n = 3. EC_50_ results n = 3.^b^ATCC BAA-1382.^c^ATCC 43255.^d^M7404.^e^ATCC BAA-1803.^f^CD26A54_S.^g^CD26A54_R.^h^*S. aureus* MRSA (ATCC 43300), *E. coli* (ATCC 25922), *K. pneumoniae* (ATCC 700603), *A. baumannii* (ATCC 19606) and *P. aeruginosa* (ATCC 27853).

**Table 2:** EC_50_ of selected Mtz-triazoles against *G. lamblia* strains. The EC_50_ values for *G. lamblia* WB strain are shown from Table 3 for comparison.

Compound		EC_50_ (μM)			
		*G. lamblia*			
		WB	106	713	713M
		MtzS	MtzS	MtzS	MtzR
**1**	metronidazole	7.9	2.8	2.3	17
**4a**	phenyl	0.25	0.28	0.16	2.3
**4b**	p-OMe-phenyl	0.27	0.64	0.64	1.0
**4c**	p-Cl-phenyl	0.20	0.89	1.2	3.5
**4d**	p,m-Cl_2_-phenyl	0.39	1.1	2.5	1.2
**4e**	p-Me-phenyl	0.18	0.29	0.29	1.0
**4f**	methyl ester	3.0	2.0	1.8	3.1
**4h**	pyridine	0.74	0.38	0.28	0.95
**4k**	benzyl	0.70	0.51	0.32	1.1
**4l**	CHOH-phenyl	4.4	2.2	2.4	3.0
**4m**	CH_2_NMe-benzyl	1.6	1.1	0.71	2.2
**4n**	thiophene	0.37	0.34	0.18	0.9

**Table 3:** MIC of selected Mtz-triazoles against *H. pylori* strains.

Compound		MIC (μg/mL)			
		*H. pylori*			
		26695	13/25^a^	98/285^a^	13/61^a^
		MtzS	MtzS	MtzR	MtzR
**1**	metronidazole	8	4-8	64	32-64
**4f**	methyl ester	32-64	32-64	64->64	64->64
**4g**	pyrazole	2-4	2	16-32	16-32
**4h**	pyridine	2	1	32	16
**4m**	CH_2_NMe-benzyl	4	2	16->64	16
**4s**	COOH	>64	>64	>64	>64

a clinical isolate.
