# Supplementary material for: Metronidazole-triazole conjugates: Activity against Clostridium difficile and parasites
Source: Eur J Med Chem. 2015 Aug 28;101:96–102. doi: 10.1016/j.ejmech.2015.06.019 (PMC4550478; doi:10.1016/j.ejmech.2015.06.019)
Supplement: Supplementary file 1 [file mmc1.docx]

Supplementary Information

Metronidazole-triazole conjugates: activity against *Clostridium difficile* and parasites

Angie M. Jarrad, Tomislav Karoli, Anjan Debnath, Chin Y. Tay, Johnny X. Huang, Geraldine Kaeslin, Alysha G. Elliott, Yukiko Miyamoto, Soumya Ramu, Angela M. Kavanagh, J. Zuegg, Lars Eckmann, Mark A.T. Blaskovich and Matthew A. Cooper

Table of Contents

1 General Methods 2

2 Synthesis 3

3 Spectra 12

4 Biological Methods 61

4.1 Determination of Minimum Inhibition Concentration (MIC) against facultative anaerobic bacteria 61

4.2 Maintenance of *C. difficile*^5^ 61

4.3 Determination of Minimum Inhibition Concentration (MIC) against *C. difficile* 62

4.4 Determination of Minimum Inhibition Concentration (MIC) by E-test against *C. difficile* 62

4.5 Determination of Minimum Inhibition Concentration (MIC) against *H. pylori* 62

*4.6* Maintenance of *E. histolytica* and *G. lamblia* 63

4.7 Determination of EC_50_ against *E. histolytica* and *G. lamblia* trophozoites 63

4.8 Cytotoxicity 63

4.9 Correlation analysis of compound activity and properties 64

5 Supplementary Results: Tables S1-3, Figure S1 65

5.1 MIC results of *Clostridium difficile* at 24 and 48 hr 65

5.2 Metronidazole E-test 66

5.3 Comparison of purified and crude Mtz-triazole activity against *G. lamblia* strains 66

5.4 Correlation matrix of compound activity and properties 66

6 References 68

## General Methods

Reagents and anhydrous solvents (dimethylformamide, dichloromethane, and acetonitrile) were used as received. Reactions requiring anhydrous conditions were performed under an inert atmosphere of nitrogen. Reactions were monitored by thin layer chromatography (TLC) or LCMS. Analytical TLC was performed on Merck TLC aluminium sheets pre-coated with Silica Gel 60 F-254 and compounds were visualised using UV lamp and potassium permanganate stain. Melting points were determined using a Gallenkamp melting point apparatus and are corrected to a standard curve of the measured and literature melting points of vanillin, acetyl salicylic acid, 3-phenoxybenzoic acid and caffeine standards. Analytical LCMS was performed on Shimadzu LCMS using 0.05% formic acid in water as solvent A and 0.05% formic acid in acetonitrile as solvent B. LCMS conditions (solvent A = H_2_O + 0.05% formic acid, solvent B = acetonitrile + 0.05% formic acid): Standard (unspecified): Column Zorbax Eclipse XDB-Phenyl, 3.0×100mm, 3.5 μ: Flow: 1 mL/min: Gradient timetable: 0.00 min, 5% B; 3.00 min, 100% B; 3.7 min, 100% B; 5.00 min, 5% B. Waters Column: Waters Atlantis T3, 2.1×50mm, 5 μ: Flow 1 mL/min, Gradient timetable: 0.00 min, 0% B; 3.30 min, 25% B; 3.50 min, 100% B; 4.00 min 100% B; 5.00 min, 0% B; 13.00 min, 5.0% B. Detection: UV at 254 nm, ELSD and electrospray MS. Compounds were purified using Biotage Isolera, Gilson or Grace Reveleris X2 chromatography systems. Commercially available cartridges were used for MPLC chromatography: Biotage SNAP cartridge HP-Silica 10, 25 or 50 g, Reveleris 4 g Silica (40 µm) cartridge or Reveleris C18 Reversed-Phase 12 g cartridge. All final products were obtained in >95% purity as determined by HPLC using UV at 254 nm, ESIMS and ELSD detection. NMR data were collected and calibrated in DMSO-*d*_6_ or CDCl_3_ at 298K on a Varian Unity 400 MHz or Bruker Avance-600 MHz spectrometers. Where appropriate, ^1^H-coupling constants were examined using resolution enhancement with MestReNova software. Data are presented as follows: chemical shift (ppm), multiplicity (s = singlet, d = doublet, t= triplet, q = quartet, quin = quintet, m = multiplet, br = broad), coupling constant (Hz) and integration. High resolution mass spectrometry (HRMS) was performed on a Bruker MicroTOF mass spectrometer using (+)-ESI calibrated to HCOONa. For compounds purified by reverse phase chromatography, the exact concentration of the compounds was determined by the quantitative NMR integration ‘PULCON’ experiment.^1^ These settings were used for all PULCON experiments: relaxation delay of 30 s, 8 scans, 2 dummy scans, 90° pulse and temperature at 298 K. The formula weight obtained from PULCON experiments was also used to calculate the percentage yields.

## Synthesis

2-(2-methyl-5-nitro-1H-imidazol-1-yl)ethyl methanesulfonate; **2**

Methanesulfonyl chloride (2.7 mL, 35.0 mmol) was added dropwise to a stirring suspension of metronidazole (4.99 g, 29.2 mmol) and TEA (6.1 mL, 43.7 mmol) in anh. DCM (25 mL) cooled to 2 °C. The reaction was warmed to room temperature and stirred for 1 hr 20 min. Volatiles were removed in vacuo. The solid was washed with water (40 mL), triturated with pet. spirits (20 mL) and washed with DCM (15 mL) and then dried under vacuum. An off-white solid was obtained (6.18 g, 85%). LCMS: R_t_ = 2.28 min, 99 A% @ 254 nm, [M + H]^+^ = 249.8. ^1^H NMR (600 MHz, DMSO-*d*_6_) δ 8.06 (s, 1H), 4.65 (dd, *J* = 5.4, 4.5 Hz, 2H), 4.55 (dd, *J* = 5.4, 4.4 Hz, 2H), 3.15 (s, 3H), 2.46 (s, 3H). ^13^C NMR (150 MHz, DMSO-*d*_6_) δ 150.7, 138.4, 133.1, 68.4, 45.1, 36.7, 14.0. HRMS calcd for C_7_H_11_N_3_NaO_5_S [M + Na]^+^, 272.0312; found, 272.0312.

1-(2-azidoethyl)-2-methyl-5-nitro-1H-imidazole; **3**

To stirring suspension of mesylate (4.50 g, 18.1 mmol) in anh. DMF (45 mL) was added NaN­­_3_ (1.48 g, 22.8 mmol). The reaction was stirred under N_2_ at 50 ˚C overnight before pouring into H_2_O (450 mL) and extracting with EtOAc (3 x 360 mL). The combined organic layers were washed with brine, dried over MgSO_4_, filtered and volatiles removed in vacuo to give an orange solid (3.60 g, 100%). LCMS: Rt = 2.50 min, 99 A% @ 254 nm, [M + H]^+^ = 197.1. ^1^H NMR (600 MHz, DMSO-*d*_6_) δ 8.06 (s, 1H), 4.48 (dd, *J* = 6.1, 5.3 Hz, 2H), 3.79 (dd, *J* = 6.1, 5.3 Hz, 2H), 2.48 (s, 3H). ^13^C NMR (150 MHz, DMSO-*d*_6_) δ 151.5, 138.3, 133.1, 50.0, 44.9, 14.0. HRMS calcd for C_6_H_8_N_6_NaO_2_ [M + Na]^+^, 219.0601; found, 219.0590.

Triazole General Procedure A:

To a stirring solution of **3** (1 eq.) in MeOH (5 vol) was added alkyne (1 eq.) followed by 100 mM aq. CuSO_4_ (5 mol %) and 100 mM aq. sodium ascorbate (10 mol %).

Triazole General Procedure B:

As for General procedure A, except that if the reaction was not proceeding when monitored by LCMS, the reaction was warmed to 45 °C and an additional portion of methanol (5 vol) was added.

Triazole General Procedure C:
As for General procedure A, except that if the reaction was not proceeding when monitored by LCMS, further portions of 100 mM aq. CuSO_4_ (5 mol %) and 100 mM aq. sodium ascorbate (10 mol %) were added.

1-(2-(2-methyl-5-nitro-1H-imidazol-1-yl)ethyl)-4-phenyl-1H-1,2,3-triazole; **4a**

General procedure A. The reaction mixture was concentrated to dryness and purified by MPLC over silica gel (Biotage, 75- 100% EtOAc in pet. spirits then 0-10% MeOH in EtOAc) to give a colourless solid (21 mg, 68%). LCMS: R_t_ = 2.51 min, 99 A% @ 254 nm, [M+ ACN + H]^+^ = 340.0. ^1^H NMR (600 MHz, DMSO-*d*_6_) δ 8.44 (s, 1H), 8.07 (s, 1H), 7.86 – 7.62 (m, 2H), 7.49 – 7.41 (m, 2H), 7.39 – 7.30 (m, 2H), 4.88 (dd, *J* = 7.1, 6.1 Hz, 2H), 4.77 (dd, *J* = 6.9, 5.8 Hz, 2H), 1.92 (s, 3H). ^13^C NMR (150 MHz, DMSO-*d*_6_) δ 151.2, 146.5, 138.4, 133.3, 130.3, 128.9, 128.0, 125.1, 122.2, 48.9, 46.1, 12.9. HRMS calcd for C_14_H_14_N_6_NaO_2_ [M + Na]^+^, 321.1070; found, 321.1063.

1-(2-(2-methyl-5-nitro-1H-imidazol-1-yl)ethyl)-4-(4-methoxyphenyl)-1H-1,2,3-triazole; **4b**

General procedure A. The reaction mixture was concentrated to dryness and purified by MPLC over silica gel (Gilson, 100% EtOAc to 30% MeOH) to give a colourless solid (71 mg, 42%). LCMS: R_t_ = 2.50 min, 99 A% @ 254 nm, [M + H]^+^ = 329.3. ^1^H NMR (600 MHz, DMSO-*d_6_*) δ 8.31 (s, 1H), 8.06 (s, 1H), 7.71 – 7.65 (m, 2H), 7.04 – 6.98 (m, 2H), 4.85 (dd, *J* = 6.5, 4.5 Hz, 2H), 4.76 (dd, *J* = 6.6, 4.4 Hz, 2H), 3.78 (s, 3H), 1.91 (s, 3H). ^13^C NMR (150 MHz, DMSO-*d_6_*) δ 159.1, 151.2, 146.5, 138.4, 133.3, 126.5, 122.9, 121.3, 114.3, 55.1, 48.8, 46.1, 12.9. HRMS calcd for C_15_H_16_N_6_NaO_3_ [M + Na]^+^, 351.1176; found, 351.1178.

1-(2-(2-methyl-5-nitro-1H-imidazol-1-yl)ethyl)-4-(4-chlorophenyl)-1H-1,2,3-triazole; **4c**

General procedure B. The reaction mixture was concentrated to dryness and purified by MPLC over silica gel (Biotage, 0- 10% MeOH in DCM) to obtain a colourless solid (82 mg, 48%). LCMS: R_t_ = 2.69 min, 99 A% @ 254 nm, [M + H]^+^ = 332.9. ^1^H NMR (600 MHz, DMSO-*d_6_*) δ 8.49 (s, 1H), 8.06 (s, 1H), 7.81 – 7.75 (m, 2H), 7.54 – 7.49 (m, 2H), 4.88 (dd, *J* = 6.4, 4.7 Hz, 2H), 4.77 (dd, *J* = 6.6, 4.6 Hz, 2H), 1.94 (s, 3H). ^13^C NMR (150 MHz, DMSO-*d_6_*) δ 151.2, 145.4, 138.3, 133.3, 132.4, 129.2, 129.0, 126.8, 122.6, 48.9, 46.0, 12.9. HRMS calcd for C_14_H_13_ClN_6_NaO_2_ [M + Na]^+^, 355.0681; found, 355.0682.

1-(2-(2-methyl-5-nitro-1H-imidazol-1-yl)ethyl)-4-(3,4-dichlorophenyl)-1H-1,2,3-triazole; **4d**

General procedure B. The reaction mixture was concentrated to dryness and purified by MPLC over silica gel (Biotage, 0-10% MeOH in DCM) to obtain a colourless pearlescent solid (129 mg, 69%). LCMS: R_t_ = 2.86 min, 99 A% @ 254 nm, [M + H]^+^ = 366.9. ^1^H NMR (600 MHz, DMSO-*d_6_*) δ 8.59 (s, 1H), 8.06 (s, 1H), 8.02 (d, *J* = 2.0 Hz, 1H), 7.77 (dd, *J* = 8.4, 2.0 Hz, 1H), 7.72 (d, *J* = 8.3 Hz, 1H), 4.89 (dd, *J* = 6.5, 4.7 Hz, 2H), 4.77 (dd, *J* = 6.5, 4.7 Hz, 2H), 1.96 (s, 3H). ^13^C NMR (150 MHz, DMSO-*d_6_*) δ 151.2, 144.3, 138.3, 133.3, 131.7, 131.3, 131.0, 130.3, 126.7, 125.1, 123.3, 49.0, 45.9, 13.0. HRMS calcd for C_14_H_12_Cl_2_N_6_NaO_2_ [M + Na]^+^, 389.0291; found, 389.0292.

1-(2-(2-methyl-5-nitro-1H-imidazol-1-yl)ethyl)-4-(p-tolyl)-1H-1,2,3-triazole; **4e**

General procedure A. The reaction mixture was concentrated to dryness and purified by MPLC over silica gel (Biotage, 0- 10% MeOH in DCM gradient) to give a colourless solid (129 mg, 81%). LCMS: R_t_ = 2.62 min, 99 A% @ 254 nm, [M + H]^+^ = 313.0. ^1^H NMR (600 MHz, DMSO-*d*_6_) δ 8.37 (s, 1H), 8.06 (s, 1H), 7.67 – 7.61 (m, 2H), 7.27 – 7.22 (m, 2H), 4.86 (dd, *J* = 6.5, 4.5 Hz, 2H), 4.76 (dd, *J* = 6.6, 4.5 Hz, 2H), 2.32 (s, 3H), 1.92 (s, 3H). ^13^C NMR (150 MHz, DMSO-*d*_6_) δ 151.2, 146.6, 138.3, 137.3, 133.2, 129.4, 127.6, 125.0, 121.8, 48.8, 46.0, 20.8, 12.9. HRMS calcd for C_15_H_16_N_6_NaO_2_ [M + Na]^+^, 335.1227; found, 335.1222.

1-(2-(2-methyl-5-nitro-1H-imidazol-1-yl)ethyl)-4-(methoxycarbonyl)- 1H-1,2,3-triazole; **4f**

General procedure A. The reaction mixture was concentrated to dryness and purified by MPLC over silica gel (Biotage, 2-10% MeOH in DCM gradient and then Grace Reveleris X2, 2-10% MeOH in DCM gradient). The product crystallised from a fraction and was collected by filtration to give a colourless solid (43 mg, 30%). Mp = 197 - 198 °C (decomposed). LCMS: R_t_ = 2.02 min, 99 A% @ 254 nm, [M + H]^+^ = 280.8. ^1^H NMR (600 MHz, DMSO-*d*_6_) δ 8.71 (s, 1H), 8.05 (s, 1H), 4.90 (dd, *J* = 6.5, 4.4 Hz, 2H), 4.77 (dd, *J* = 6.6, 4.5 Hz, 2H), 3.82 (s, 3H), 1.93 (s, 3H). ^13^C NMR (150 MHz, DMSO-*d*_6_) δ 160.4, 151.0, 138.6, 138.4, 133.2, 130.0, 51.8, 49.1, 45.7, 12.9. HRMS calcd for C_10_H_12_N_6_NaO_4_ [M + Na]^+^, 303.0812; found, 303.0814.

1-(2-(2-methyl-5-nitro-1H-imidazol-1-yl)ethyl)-4-((1H-pyrazol-1-yl)methyl)-1H-1,2,3-triazole; **4g**

General procedure A. The reaction mixture was concentrated to dryness and the residue was purified by MPLC over silica gel (Biotage, 2- 15% MeOH in DCM gradient). The product was obtained as a colourless solid (57 mg, 55%). LCMS: R_t_ = 2.03 min, 99 A% @ 254 nm, [M + H]^+^ = 302.9. ^1^H NMR (600 MHz, DMSO-*d*_6_) δ 8.03 (s, 1H), 7.95 (s, 1H), 7.71 (dd, *J* = 2.3, 0.7 Hz, 1H), 7.42 (dd, *J* = 1.9, 0.7 Hz, 1H), 6.24 (t, *J* = 2.1 Hz, 1H), 5.37 (s, 2H), 4.81 (dd, *J* = 6.5, 4.4 Hz, 2H), 4.69 (dd, *J* = 6.5, 4.5 Hz, 2H), 1.79 (s, 3H). ^13^C NMR (150 MHz, DMSO*-d*_6_) δ 151.2, 143.3, 138.9, 138.3, 133.2, 129.6, 124.6, 105.4, 48.7, 46.2, 46.1, 12.7. HRMS calcd for C12H14N8NaO2 [M + Na]^+^, 325.1132; found, 325.1129.

1-(2-(2-methyl-5-nitro-1H-imidazol-1-yl)ethyl)-4-(2-pyridinyl)-1H-1,2,3-triazole.xTFA; **4h**

General procedure A. The crude mixture was diluted with water and the organics were extracted with DCM (3 x 50 mL). The combined organics were washed with brine, dried over anh. MgSO_4_ and filtered. Volatiles were removed *in vacuo* and the residue was purified by MPLC over C18 silica gel (Grace Reveleris X2, A: H_2_O + 0.1% TFA, B: ACN + 0.1% TFA, 5-30% B). The eluent was removed by lyophilisation to give a yellowish solid (148 mg, 97%). LCMS: R_t_ = 2.08 min, 99 A% @ 254 nm, [M + H]^+^ = 299.8. ^1^H NMR (600 MHz, DMSO-*d*_6_) δ 8.59 (ddd, *J* = 5.0, 1.8, 1.0 Hz, 1H), 8.55 (s, 1H), 8.06 (s, 1H), 8.03 (dt, *J* = 8.0, 1.1 Hz, 1H), 7.92 (td, *J* = 7.7, 1.8 Hz, 1H), 7.38 (ddd, *J* = 7.6, 4.9, 1.2 Hz, 1H), 4.92 (dd, *J* = 6.5, 4.4 Hz, 2H), 4.80 (dd, *J* = 6.5, 4.4 Hz, 2H), 1.93 (s, 3H). ^13^C NMR (150 MHz, DMSO- *d*_6_) δ 158.5, 158.3, 158.0, 157.8, 151.1, 149.3, 147.0, 138.4, 137.7, 133.1, 124.4, 123.2, 119.6, 49.0, 46.0, 12.9. HRMS calcd for C_13_H_14_N_7_O_2_ [M + H]^+^, 300.1203; found, 300.1194.

1-(2-(2-methyl-5-nitro-1H-imidazol-1-yl)ethyl)-4-(5-pyrimidinyl)-1H-1,2,3-triazole; **4i**

General procedure A. The reaction was diluted with DCM (10 mL). The tan ppt was then collected by filtration. The organic layer filtrate was evaporated to give a crude crystalline solid. The ppt and crystals were combined and purified by MPLC over C18 silica gel (Grace Reveleris X2, A: H_2_O + 0.1% TFA, B: ACN + 0.1% TFA, 5-30% B). The eluent was removed by lyophilisation to give a colourless powder (86 mg, 56%). LCMS: R_t_ = 1.99 min, 99 A% @ 254 nm, [M + H]^+^ = 300.8. ^1^H NMR (600 MHz, DMSO-*d*_6_) δ 9.19 (s, 2H), 9.18 (s, 1H), 8.70 (s, 1H), 8.07 (s, 1H), 4.94 (dd, *J* = 6.5, 4.7 Hz, 2H), 4.79 (dd, *J* = 6.5, 4.8 Hz, 2H), 2.00 (s, 3H). ^13^C NMR (150 MHz, DMSO-*d*_6_) δ 157.7, 153.3, 151.3, 140.8, 138.4, 133.3, 124.7, 123.7, 49.1, 45.9, 13.0. HRMS calcd for C_12_H_12_N_8_NaO_2_ [M + Na]^+^, 323.0975; found, 323.0966.

1-(2-(2-methyl-5-nitro-1H-imidazol-1-yl)ethyl)-4-(2-pyrimidinyl)-1H-1,2,3-triazole; **4j**

General procedure A. The reaction was poured into water (20 mL) and extracted with DCM (4 x 50 mL). The combined organics were washed with brine (50 mL), dried over anh. MgSO_4_ and filtered. The volatiles were removed *in vacuo* and the crude was purified by MPLC over C18 silica gel (Grace Reveleris X2, A: H_2_O + 0.1% TFA, B: ACN + 0.1% TFA, 5-25%) then repurified (Grace Reveleris X2, A: H_2_O + 0.1% TFA, B: ACN + 0.1% TFA, 5-100% B) to give a cream powder (16 mg, 9%). LCMS: R_t_ = 2.21 min, 99 A% @ 254 nm, [M + H]^+^ = 300.8. ^1^H NMR (600 MHz, DMSO-*d*_6_) δ 8.88 – 8.84 (m, 2H), 8.69 (s, 1H), 8.06 (s, 1H), 7.44 (t, *J* = 4.9 Hz, 1H), 4.93 (dd, *J* = 6.5, 4.4 Hz, 2H), 4.80 (dd, *J* = 6.6, 4.4 Hz, 2H), 1.93 (s, 3H). ^13^C NMR (150 MHz, DMSO-*d*_6_) δ 158.3, 157.8, 151.1, 146.5, 138.4, 133.2, 127.3, 120.2, 48.9, 46.0, 12.9. HRMS calcd for C_12_H_12_N_8_NaO_2_ [M + Na]^+^, 323.0975; found, 323.0976.

1-(2-(2-methyl-5-nitro-1H-imidazol-1-yl)ethyl)- 4-benzyl-1H-1,2,3-triazole; **4k**

General procedure A. The reaction mixture was concentrated to dryness and purified by MPLC over silica gel (Biotage, 0- 10% MeOH in DCM gradient) to give a colourless solid (34 mg, 21%). LCMS: R_t_ = 2.50 min, 99 A% @ 254 nm, [M + H]^+^ = 313.0. ^1^H NMR (600 MHz, DMSO-*d*_6_) δ 8.03 (s, 1H), 7.69 (t, *J* = 0.7 Hz, 1H), 7.32 – 7.25 (m, 2H), 7.23 – 7.15 (m, 3H), 4.77 (dd, *J* = 6.5, 4.3 Hz, 2H), 4.68 (dd, *J* = 6.5, 4.4 Hz, 2H), 3.95 (s, 2H), 1.80 (s, 3H). ^13^C NMR (150 MHz, DMSO-*d*_6_) δ 151.1, 146.4, 139.4, 138.3, 133.2, 128.4, 128.3, 126.1, 123.4, 48.5, 46.2, 31.0, 12.8. HRMS calcd for C_15_H_16_N_6_NaO_2_ [M + Na]^+^, 335.1227; found, 335.1224.

1-(2-(2-methyl-5-nitro-1H-imidazol-1-yl)ethyl)-4-(1-phenyl-1-hydroxylmethyl)- 1H-1,2,3-triazole; **4l**

General procedure A. The reaction mixture was concentrated to dryness and purified by MPLC over silica gel (Biotage, 0- 10% MeOH in DCM gradient) to give an off-white solid which was recrystallised (EtOAc) to yield an off-white crystalline solid (75 mg, 45%). Mp = 136-137 °C. LCMS: R_t_ = 2.19 min, 99 A% @ 254 nm, [M + H]^+^ = 328.9. ^1^H NMR (600 MHz, DMSO-*d*_6_) δ 8.03 (s, 1H), 7.77 – 7.74 (m, 1H), 7.35 – 7.28 (m, 4H), 7.28 – 7.21 (m, 1H), 5.96 (d, *J* = 4.7 Hz, 1H), 5.77 (d, *J* = 4.7 Hz, 1H), 4.83 – 4.73 (m, 2H), 4.73 – 4.61 (m, 2H), 1.75 (s, 3H). ^13^C NMR (150 MHz, DMSO-*d*_6_) δ 151.9, 151.2, 143.9, 138.3, 133.2, 128.0, 127.0, 126.3, 122.9, 67.8, 48.5, 46.2, 12.7. HRMS calcd for C_15_H_16_N_6_NaO_3_ [M + Na]^+^, 351.1176; found, 351.1173.

1-(2-(2-methyl-5-nitro-1H-imidazol-1-yl)ethyl)-4-(N-benzyl, N-methyl aminomethyl)- 1H-1,2,3-triazole; **4m**

General procedure A. The reaction mixture was concentrated to dryness and purified by MPLC over silica gel (Biotage, 0- 20% MeOH in DCM gradient, recolumned 0- 15% MeOH in DCM) to give an orange waxy solid (77 mg, 42%). LCMS: R_t_ = 1.88 min, 99 A% @ 254 nm, [M + H]^+^ = 356.1. ^1^H NMR (600 MHz, DMSO-*d*_6_) δ 8.03 (s, 1H), 7.89 (s, 1H), 7.35 – 7.27 (m, 4H), 7.27 – 7.22 (m, 1H), 4.85 – 4.80 (m, 2H), 4.76 – 4.71 (m, 2H), 3.57 (s, 2H), 3.42 (s, 2H), 2.05 (s, 3H), 1.87 (s, 3H). ^13^C NMR (150 MHz, DMSO-*d*_6_) δ 151.1, 143.7, 138.7, 138.3, 133.2, 128.6, 128.1, 126.8, 124.6, 60.0, 51.0, 48.6, 46.2, 41.2, 12.9. HRMS calcd for C_17_H_22_N_7_O_2_ [M + H]^+^, 356.1829; found, 356.1824.

1-(2-(2-methyl-5-nitro-1H-imidazol-1-yl)ethyl)-4-(thiophen-3-yl)-1H-1,2,3-triazole; **4n**

General procedure A. The reaction mixture was concentrated to dryness and purified by MPLC over C18 silica gel (Grace Reveleris X2, A: H_2_O + 0.1% TFA, B: ACN + 0.1% TFA, 20-50% B). The eluent was removed by lyophilisation to give a colourless powdery solid (22.4 mg, 14%). LCMS: R_t_ = 2.43 min, 99 A% @ 254 nm, [M + H]^+^ = 305.1. ^1^H NMR (600 MHz, DMSO-*d*_6_) δ 8.30 (s, 1H), 8.06 (s, 1H), 7.80 (dd, *J* = 3.0, 1.3 Hz, 1H), 7.64 (dd, *J* = 5.0, 2.9 Hz, 1H), 7.43 (dd, *J* = 5.0, 1.3 Hz, 1H), 4.86 (dd, *J* = 6.6, 4.5 Hz, 2H), 4.76 (dd, *J* = 6.6, 4.5 Hz, 2H), 1.93 (s, 3H). ^13^C NMR (150 MHz, DMSO-*d*_6_) δ 151.2, 143.0, 138.3, 133.3, 131.6, 127.2, 125.6, 121.9, 121.0, 48.8, 46.0, 12.9. HRMS calcd for C_12_H_12_N_6_NaO_2_S [M + Na]^+^, 327.0635; found, 327.0627.

1-(2-(2-methyl-5-nitro-1H-imidazol-1-yl)ethyl)-4-((1,1-dioxothiomorpholin-4-yl)methyl)- 1H-1,2,3-triazole; **4o**

General procedure C. The reaction was evaporated directly onto C18 silica and purified by MPLC over C18 silica gel (Grace Reveleris X2, A: H_2_O + 0.1% TFA, B: ACN + 0.1% TFA, 5-10%). The eluent was removed by lyophilisation to give an aqua coloured powder. The solid was dissolved in H_2_O (5 mL), adjusted to pH 5 with 2M aq. NaOH and passed over Chelex 100 resin, 200-400 mesh. The eluent was removed by lyophilisation to give a tan solid (93 mg, 43%). LCMS: R_t_ = 1.85 min, 99 A% @ 254 nm, [M + H]^+^ = 370.0. ^1^H NMR (600 MHz, DMSO-*d*_6_) δ 8.05 (s, 1H), 7.88 (s, 1H), 4.82 (dd, *J* = 6.6, 4.3 Hz, 2H), 4.72 (dd, *J* = 6.6, 4.3 Hz, 2H), 3.72 (s, 2H), 3.10 – 3.05 (m, 4H), 2.86 – 2.80 (m, 4H), 1.87 (s, 3H). ^13^C NMR (150 MHz, DMSO-*d*_6_) δ 151.2, 143.2, 138.3, 133.2, 124.8, 50.5, 50.5, 49.7, 48.7, 46.1, 12.9. HRMS calcd for C_13_H_19_N_7_NaO_4_S [M + Na]^+^, 392.1111; found, 392.1110.

1-(2-(2-methyl-5-nitro-1H-imidazol-1-yl)ethyl)-4-(2-hydroxyethyl)-1H-1,2,3-triazole; **4p**

**

General procedure B and C. An additional portion of acetylene (1 eq.) was added. The crude reaction mixture was concentrated to dryness and purified by MPLC over silica gel (Biotage, 15-25% MeOH in DCM) to give a colourless solid (49 mg, 68%). LCMS: R_t_ = 1.73 min, 99 A% @ 254 nm, [M + H]^+^ = 267.1. ^1^H NMR (600 MHz, DMSO-*d*_6_) δ 8.04 (s, 1H), 7.74 (s, 1H), 4.80 – 4.75 (m, 2H), 4.71 – 4.65 (m, 3H), 3.58 – 3.52 (m, 2H), 2.75 – 2.69 (m, 2H), 1.84 (s, 3H). ^13^C NMR (150 MHz, DMSO-*d*_6_) δ 151.2, 144.7, 138.3, 133.2, 123.3, 60.4, 48.5, 46.2, 29.0, 12.7. HRMS calcd for C_10_H_14_N_6_NaO_3_ [M + Na]^+^, 289.1020; found, 289.1013.

1-(2-(2-methyl-5-nitro-1H-imidazol-1-yl)ethyl)-4-(hydroxymethyl)- 1H-1,2,3-triazole; **4q**

**

General procedure B and C. An additional portion of acetylene (1 eq.) was added. The crude reaction mixture was concentrated to dryness and purified by MPLC over silica gel (Biotage, 2-15% MeOH in DCM) to give a colourless solid (34 mg, 51%). LCMS: R_t_ = 1.67 min, 99 A% @ 254 nm, [M + H]^+^ = 253.0. ^1^H NMR (600 MHz, DMSO-*d*_6_) δ 8.05 (s, 1H), 7.87 (s, 1H), 5.20 (t, *J* = 5.6 Hz, 1H), 4.81 (dd, *J* = 6.5, 4.4 Hz, 2H), 4.71 (dd, *J* = 6.5, 4.4 Hz, 2H), 4.49 – 4.45 (m, 2H), 1.85 (s, 3H). ^13^C NMR (150 MHz, DMSO-*d*_6_) δ 151.2, 148.3, 138.3, 133.2, 123.6, 54.8, 48.5, 46.2, 12.8. HRMS calcd for C_9_H_12_N_6_NaO_3_ [M + Na]^+^, 275.0863; found, 275.0865.

sodium 1-(2-(2-methyl-5-nitro-1H-imidazol-1-yl)ethyl)-4-(carboxyethyl)- 1H-1,2,3-triazole; **4r**

General procedure C. The reaction was evaporated directly onto C18 silica and purified by MPLC over C18 silica gel (Grace Reveleris X2, A: H_2_O + 0.1% TFA, B: ACN + 0.1% TFA, 5-14% B). The eluent was removed by lyophilisation to give an aqua coloured powder. The solid was dissolved in ACN: H_2_O (30:70, 20 mL), adjusted to pH 5 with 2M aq. NaOH and passed over Chelex 100 resin, 200-400 mesh. The eluent was removed by lyophilisation to give a tan powder (109 mg, 73%). LCMS: R_t_ = 1.82 min, 99 A% @ 254 nm, [M + H]^+^ = 294.7. ^1^H NMR (600 MHz, DMSO-*d*_6_) δ 8.04 (s, 1H), 7.70 (s, 1H), 4.78 – 4.73 (m, 2H), 4.70 – 4.65 (m, 2H), 2.76 (br s, 2H), 2.36 (br s, 2H), 1.82 (br s, 3H). ^13^C NMR (150 MHz, DMSO*-d*_6_) δ 175.0*, 151.2, 147.0, 138.3, 133.2, 122.7, 48.27, 46.21, 35.8*, 21.46, 12.8. *Carbon peaks were confirmed by HSQC and HMBC correlations. HRMS calcd for C_11_H_14_N_6_NaO_4_ [M + Na]^+^, 317.0969; found, 317.0958.

1-(2-(2-methyl-5-nitro-1H-imidazol-1-yl)ethyl)-4-(carboxy)- 1H-1,2,3-triazole; **4s**

**

General procedure A. The reaction suspension was filtered. The solid was washed with water (5 x 0.5 mL) and EtOAc (3 x 0.5 mL). The solid was purified by MPLC over C18 silica gel (Grace Reveleris X2, A: H_2_O + 0.1% TFA, B: ACN + 0.1% TFA, 5-15%). The eluent was removed by lyophilisation to give a colourless solid (49 mg, 36%). LCMS: R_t_ = 1.72 min, 99 A% @ 254 nm, [M + H]^+^ = 266.8. ^1^H NMR (600 MHz, DMSO-*d*_6_) δ 8.59 (s, 1H), 8.05 (s, 1H), 4.89 (dd, *J* = 6.5, 4.4 Hz, 2H), 4.76 (dd, *J* = 6.6, 4.4 Hz, 2H), 1.93 (s, 3H). ^13^C NMR (150 MHz, DMSO-*d*_6_) δ 161.4, 151.0, 139.7, 138.4, 133.2, 129.8, 49.0, 45.8, 12.9. HRMS calcd for C_9_H_10_N_6_NaO_4_ [M + Na]^+^, 289.0656; found, 289.0655.

1-(2-(2-methyl-5-nitro-1H-imidazol-1-yl)ethyl)-4-(aminomethyl)- 1H-1,2,3-triazole trifluoroacetate salt; **4t**

General procedure A. The reaction was evaporated directly onto C18 silica and purified by MPLC over C18 silica gel (Grace Reveleris X2, A: H_2_O + 0.1% TFA, B: ACN + 0.1% TFA, 5-10% B). The eluent was removed by lyophilisation to give a yellow oil (34 mg, 17%). LCMS: R_t_ = 1.26 min, 99 A% @ 254 nm, [M^+^H]^+^ = 251.8. ^1^H NMR (600 MHz, DMSO-*d*_6_) δ 8.24 (br s, 3H), 8.072 (s, 1H), 8.068 (s, 1H), 4.89 (dd, *J* = 6.5, 4.5 Hz, 2H), 4.74 (dd, *J* = 6.6, 4.6 Hz, 2H), 4.11 (q, *J* = 5.8 Hz, 2H), 1.92 (s, 3H). ^13^C NMR (150 MHz, DMSO*-d*_6_) δ 151.3, 140.3, 138.4, 133.2, 125.1, 48.8, 46.0, 33.7, 13.0. HRMS calcd for C_9_H_14_N_7_O_2_ [M + H]^+^, 252.1203; found, 252.1204.

1-(prop-2-yn-1-yl)-1H-pyrazole; **6**

To a solid mixture of 1H-pyrazole (1.00 g, 14.7 mmol), K_2_CO_3_ (3.04 g, 22.0 mmol) and tetrabutylammonium bromide (237 mg, 0.73 mmol) was added propargyl bromide (80% weight in toluene) (2.5 mL, 22.0 mmol).^2^ The reaction was stirred vigorously at rt for 3 days before pouring into H_2_O (50 mL) and extracting with EtOAc (50 mL x 3). The combined organics were washed with brine (50 mL), dried with anh. MgSO_4_ and filtered. Volatiles removed *in vacuo* to give a crude brown oil. The crude material was purified by MPLC over silica gel (Biotage, 5- 40% EtOAc in pet. spirits gradient) to give a pale yellow oil (245 mg, 16%). LCMS: R_t_ = 1.82 min, A% @ 200 nm, [M + H]^+^ = not detected by ESI/API. ^1^H NMR (600 MHz, CDCl_3_) δ 7.62 – 7.58 (m, 1H), 7.56 – 7.52 (m, 1H), 6.30 (m, 1H), 4.96 (d, *J* = 2.6 Hz, 2H), 2.50 (t, *J* = 2.6 Hz, 1H). ^13^C NMR (150 MHz, CDCl_3_) δ 140.0, 128.7, 106.2, 76.8, 74.5, 41.5. Spectra were indistinguishable from Mohr et al.^3^

## Spectra

**2-(2-methyl-5-nitro-1H-imidazol-1-yl)ethyl methanesulfonate**

^1^H NMR (600 MHz DMSO-*d*_6_)

**2**

**2-(2-methyl-5-nitro-1H-imidazol-1-yl)ethyl methanesulfonate**

^13^C JMOD (150 MHz, DMSO-*d*_6_)

**2**

**1-(2-azidoethyl)-2-methyl-5-nitro-1H-imidazole**

^1^H NMR (600 MHz, DMSO-*d*_6_)

**3**

**1-(2-azidoethyl)-2-methyl-5-nitro-1H-imidazole**

^13^C JMOD (150 MHz, DMSO-*d*_6_)

**3**

**1-(2-(2-methyl-5-nitro-1H-imidazol-1-yl)ethyl)-4-phenyl-1H-1,2,3-triazole**

^1^H NMR (600 MHz, DMSO-*d*_6_)

**4a**

**1-(2-(2-methyl-5-nitro-1H-imidazol-1-yl)ethyl)-4-phenyl-1H-1,2,3-triazole**

^13^C JMOD (150 MHz, DMSO-*d*_6_)

**4a**

**1-(2-(2-methyl-5-nitro-1H-imidazol-1-yl)ethyl)-4-(4-methoxyphenyl)-1H-1,2,3-triazole**

^1^H NMR (600 MHz, DMSO-*d*_6_)

**4b**

**1-(2-(2-methyl-5-nitro-1H-imidazol-1-yl)ethyl)-4-(4-methoxyphenyl)-1H-1,2,3-triazole**

^13^C JMOD NMR (150 MHz, DMSO-*d*_6_)

**4b**

**1-(2-(2-methyl-5-nitro-1H-imidazol-1-yl)ethyl)-4-(4-chlorophenyl)-1H-1,2,3-triazole**

^1^H NMR (600 MHz, DMSO-*d*_6_)

**4c**

**1-(2-(2-methyl-5-nitro-1H-imidazol-1-yl)ethyl)-4-(4-chlorophenyl)-1H-1,2,3-triazole**

^13^C JMOD NMR (150 MHz, DMSO-*d*_6_)

**4c**

**1-(2-(2-methyl-5-nitro-1H-imidazol-1-yl)ethyl)-4-(3,4-dichlorophenyl)-1H-1,2,3-triazole**

^1^H NMR (600 MHz, DMSO-*d*_6_)

**4d**

**1-(2-(2-methyl-5-nitro-1H-imidazol-1-yl)ethyl)-4-(3,4-dichlorophenyl)-1H-1,2,3-triazole**

^13^C JMOD NMR (150 MHz, DMSO-*d*_6_)

**4d**

**1-(2-(2-methyl-5-nitro-1H-imidazol-1-yl)ethyl)-4-(p-tolyl)-1H-1,2,3-triazole**

^1^H NMR (600 MHz, DMSO-*d*_6_)

**4e**

**1-(2-(2-methyl-5-nitro-1H-imidazol-1-yl)ethyl)-4-(p-tolyl)-1H-1,2,3-triazole**

^13^C JMOD NMR (150 MHz, DMSO-*d*_6_)

**4e**

**1-[2-(2-methyl-5-nitro-1H-imidazol-1-yl)ethyl]-4-(methoxycarbonyl)- 1H-1,2,3-triazole**

^1^H NMR (600 MHz, DMSO-*d*_6_)

**4f**

**1-[2-(2-methyl-5-nitro-1H-imidazol-1-yl)ethyl]-4-(methoxycarbonyl)- 1H-1,2,3-triazole**

^13^C JMOD NMR (150 MHz, DMSO-*d*_6_).

**4f**

**1-(2-(2-methyl-5-nitro-1H-imidazol-1-yl)ethyl)-4-((1H-pyrazol-1-yl)methyl)-1H-1,2,3-triazole**

^1^H NMR (600 MHz, DMSO-*d*_6_)

**4g**

**1-(2-(2-methyl-5-nitro-1H-imidazol-1-yl)ethyl)-4-((1H-pyrazol-1-yl)methyl)-1H-1,2,3-triazole**

^13^C JMOD NMR (150 MHz, DMSO-*d*_6_)

**4g**

**1-(2-(2-methyl-5-nitro-1H-imidazol-1-yl)ethyl)-4-(2-pyridinyl)-1H-1,2,3-triazole.xTFA**

^1^H NMR (600 MHz, DMSO-*d*_6_)

**4h**

**1-(2-(2-methyl-5-nitro-1H-imidazol-1-yl)ethyl)-4-(2-pyridinyl)-1H-1,2,3-triazole.xTFA**

^13^C JMOD NMR (150 MHz, DMSO-*d*_6_)

**4h1-(2-(2-methyl-5-nitro-1H-imidazol-1-yl)ethyl)-4-(5-pyrimidinyl)-1H-1,2,3-triazole**

^1^H NMR (600 MHz, DMSO-*d*_6_)

**4i**

**1-(2-(2-methyl-5-nitro-1H-imidazol-1-yl)ethyl)-4-(5-pyrimidinyl)-1H-1,2,3-triazole**

^13^C JMOD NMR (150 MHz, DMSO-*d*_6_)

**4i**

**1-(2-(2-methyl-5-nitro-1H-imidazol-1-yl)ethyl)-4-(2-pyrimidinyl)-1H-1,2,3-triazole**

^1^H NMR (600 MHz, DMSO-*d*_6_)

**4j**

**1-(2-(2-methyl-5-nitro-1H-imidazol-1-yl)ethyl)-4-(2-pyrimidinyl)-1H-1,2,3-triazole**

^13^C JMOD NMR (150 MHz, DMSO-*d*_6_)

**4j**

**1-(2-(2-methyl-5-nitro-1H-imidazol-1-yl)ethyl)- 4-benzyl-1H-1,2,3-triazole**

^1^H NMR (600 MHz, DMSO-*d*_6_)

**4k**

**4-benzyl-1-(2-(2-methyl-5-nitro-1H-imidazol-1-yl)ethyl)-1H-1,2,3-triazole**

^13^C JMOD NMR (150 MHz, DMSO-*d*_6_)

**4k**

**1-(2-(2-methyl-5-nitro-1H-imidazol-1-yl)ethyl)-4-(1-phenyl-1-hydroxylmethyl)- 1H-1,2,3-triazole**

^1^H NMR (600 MHz, DMSO-*d*_6_)

**4l**

**1-(2-(2-methyl-5-nitro-1H-imidazol-1-yl)ethyl)-4-(1-phenyl-1-hydroxylmethyl)- 1H-1,2,3-triazole**

^13^C JMOD NMR (150 MHz, DMSO-*d*_6_)

**4l**

**1-(2-(2-methyl-5-nitro-1H-imidazol-1-yl)ethyl)-4-(N-benzyl, N-methyl aminomethyl)- 1H-1,2,3-triazole**

^1^H NMR (600 MHz, DMSO-*d*_6_)

**4m**

**1-(2-(2-methyl-5-nitro-1H-imidazol-1-yl)ethyl)-4-(N-benzyl, N-methyl aminomethyl)- 1H-1,2,3-triazole**

^13^C JMOD NMR (150 MHz, DMSO-*d*_6_)

**4m**

**1-(2-(2-methyl-5-nitro-1H-imidazol-1-yl)ethyl)-4-(thiophen-3-yl)-1H-1,2,3-triazole**

^1^H NMR (600 MHz, DMSO-*d*_6_)

**4n**

**1-(2-(2-methyl-5-nitro-1H-imidazol-1-yl)ethyl)-4-(thiophen-3-yl)-1H-1,2,3-triazole**

^13^C JMOD NMR (150 MHz, DMSO-*d*_6_)

**4n**

**1-(2-(2-methyl-5-nitro-1H-imidazol-1-yl)ethyl)-4-((1,1-dioxothiomorpholin-4-yl)methyl)- 1H-1,2,3-triazole**

^1^H NMR (600 MHz, DMSO-*d*_6_)

**4o**

**1-(2-(2-methyl-5-nitro-1H-imidazol-1-yl)ethyl)-4-((1,1-dioxothiomorpholin-4-yl)methyl)- 1H-1,2,3-triazole**

^13^C JMOD NMR (150 MHz, DMSO-*d*_6_)

**4o**

**1-(2-(2-methyl-5-nitro-1H-imidazol-1-yl)ethyl)-4-(2-hydroxyethyl)-1H-1,2,3-triazole**

^1^H NMR (600 MHz, DMSO-*d*_6_)

**4p**

**1-(2-(2-methyl-5-nitro-1H-imidazol-1-yl)ethyl)-4-(2-hydroxyethyl)-1H-1,2,3-triazole**

^13^C JMOD NMR (150 MHz, DMSO-*d*_6_)

**4p**

**1-(2-(2-methyl-5-nitro-1H-imidazol-1-yl)ethyl)-4-(hydroxymethyl)- 1H-1,2,3-triazole**

^1^H NMR (600 MHz, DMSO-*d*_6_)

**4q**

**1-(2-(2-methyl-5-nitro-1H-imidazol-1-yl)ethyl)-4-(hydroxymethyl)- 1H-1,2,3-triazole**

^13^C JMOD NMR (150 MHz, DMSO-*d*_6_)

**4q**

**sodium 1-(2-(2-methyl-5-nitro-1H-imidazol-1-yl)ethyl)-4-(carboxyethyl)- 1H-1,2,3-triazole**

^1^H NMR (600 MHz, DMSO-*d*_6_)

**4r**

**sodium 1-(2-(2-methyl-5-nitro-1H-imidazol-1-yl)ethyl)-4-(carboxyethyl)- 1H-1,2,3-triazole**

^13^C JMOD NMR (150 MHz, DMSO-*d*_6_). Missing carbon resonances at 35.8 ppm and 175.0 ppm were assigned with gHSQC and gHMBC correlations. **4r**

**sodium 1-(2-(2-methyl-5-nitro-1H-imidazol-1-yl)ethyl)-4-(carboxyethyl)- 1H-1,2,3-triazole**

gHSQC NMR (150 MHz, DMSO-*d*_6_).

**4r**

**sodium 1-(2-(2-methyl-5-nitro-1H-imidazol-1-yl)ethyl)-4-(carboxyethyl)- 1H-1,2,3-triazole**

gHMBC NMR (150 MHz, DMSO-*d*_6_).

**4r**

**1-(2-(2-methyl-5-nitro-1H-imidazol-1-yl)ethyl)-4-(carboxy)- 1H-1,2,3-triazole**

^1^H NMR (600 MHz, DMSO-*d*_6_)

**4s**

**1-(2-(2-methyl-5-nitro-1H-imidazol-1-yl)ethyl)-4-(carboxy)- 1H-1,2,3-triazole**

^13^C JMOD NMR (150 MHz, DMSO-*d*_6_)

**4s**

**1-(2-(2-methyl-5-nitro-1H-imidazol-1-yl)ethyl)-4-(aminomethyl)- 1H-1,2,3-triazole trifluoroacetate salt**

^1^H NMR (600 MHz, DMSO-*d*_6_)

**4t**

**1-(2-(2-methyl-5-nitro-1H-imidazol-1-yl)ethyl)-4-(aminomethyl)- 1H-1,2,3-triazole trifluoroacetate salt**

^13^C JMOD NMR (150 MHz, DMSO-*d*_6_)

**4t**

**1-(prop-2-yn-1-yl)-1H-pyrazole**

^1^H NMR (600 MHz, CDCl_3_)

**6**

**1-(prop-2-yn-1-yl)-1H-pyrazole**

^13^C JMOD (150 MHz, CDCl_3_)

**6**

## Biological Methods

### Determination of Minimum Inhibition Concentration (MIC) against facultative anaerobic bacteria

MICs were determined by a two-fold serial broth micro dilution according to the recommendation of CLSI standards with an inoculum of 5 × 10^5^ CFU/mL^4^ The compounds along with standard antibiotics were serially diluted twofold across the wells of 96-well non-binding surface plates (NBS, Corning). Standards ranged from 64 to 0.03 μg/mL, and the compounds from 128 to 0.06 μg/mL with final volumes of 50 μL per well. Gram-positive and Gram-negative bacteria were cultured in Mueller Hinton broth (MHB) (Bacto laboratories, Cat. no. 211443) at 37 °C overnight. A sample of each culture was then diluted 40-fold in fresh MHB broth and incubated at 37 °C for 2-3 h. The resultant mid-log phase cultures were diluted to the final concentration of 5 × 10^5^ CFU/mL, then 50 μL was added to each well of the compound containing 96-well plates. All the plates were covered and incubated at 37 °C for 24 h. MICs were the lowest concentration that showed no visible growth.

### Maintenance of *C. difficile*^5^

Heat shocked spore stocks in H_2_O

*C. difficile* was inoculated onto agar plates (TS + 5% sheep blood or BHIS agar) and incubated anaerobically at 37 °C for 3 to 7 days. The plates were removed from the anaerobic chamber and sterile PBS (1 mL) was added onto each agar plate. The colonies were suspended by scraping with a disposable inoculating loop. The cell suspension was transferred to an Eppendorf tube and this process was repeated with a further 0.5 mL of sterile PBS added to the agar plate. The cells were then heat shocked at 65 °C for 25 minutes to kill vegetative cells. The cells were pelleted by centrifuging at 14000 RPM for 1 minute, the supernatant was discarded and the cell pellet was resuspended in sterile PBS (1 mL). This sequence was repeated 3 times before finally suspending the spores in sterile H_2_O (1 mL) and storing at 4 °C until required.

Revival of C. difficile vegetative cells from spore stocks

*C. difficile* ATCC BAA-1382, ATCC BAA-1803, ATCC 43255 and M7404 heat shocked spore stocks (10 µL) were transferred into the anaerobic chamber and 10 µL streaked onto pre-reduced (at least 2.5 hr in anaerobic chamber) BHIS agar plates supplemented with 0.1% w/v sodium taurocholate (BHIS(TA)). Plates were incubated for 24 hours prior to assay to give colonies of size 1-3mm diameter with characteristic uneven borders.

Revival of C. difficile from Microbank beads stored at -80 °C.

*C. difficile* CD26A54_S and *C. difficile* CD26A54_R on Microbank beads were transferred into the anaerobic chamber in a -20 °C Eppendorf IsoTherm cooler system. A single bead was used to inoculate individual prereduced BAKHS agar plates (brucella agar supplemented with 10 µg/mL vitamin K_1_, 5 µg/mL hemin and 5% laked sheep blood).^6^ Inoculated agar plates were incubated anaerobically for 48 hr before subculture onto BAKHS and finally onto BHIS(TA) prior to assay. As a control inoculated agar plates were incubated aerobically and no colonies grew.

### Determination of Minimum Inhibition Concentration (MIC) against *C. difficile*

The minimum inhibition concentration was determined according to the CLSI Methods with modifications in broth and inoculum for *C. difficile* according to Babakhani *et al.*^7,8^ Briefly, compounds were prepared to 20X final concentration in 100% water, 60% DMSO and 40% water or 60% DMSO and 40% media. The compounds along with standard antibiotics were serially diluted two-fold across the wells of 96-well non-binding surface plates (Non binding surface, Corning). The plates were placed in the anaerobic chamber overnight to reduce. *C. difficile* bacteria from BHIS(TA) agar plates were cultured anaerobically in BHIS at 37 °C overnight. A sample of culture was then diluted 40-fold in BHIS broth and incubated at 37 °C for approximately 4.5 hrs. The resultant mid-log phase culture (OD_600_= 0.5-0.6) was diluted to the final concentration of ~1×10^6^ CFU/mL, then 50 µL was added to each well of the compound-containing 96-well plates. This gave final cell concentration of 5×10^5^ CFU/mL, final volume 100 µL and 3% maximum DMSO concentration. Compound concentration was typically from 64 to 0.03 µg/mL. An antibiotic standard was included on each 96 well plate as well as a positive growth control (no compound) and sterility control (no bacteria). All the plates were covered and incubated at 37 °C for 24 h. MICs were the lowest concentration showing **no visible** growth.

### Determination of Minimum Inhibition Concentration (MIC) by E-test against *C. difficile*

Mid-log phase (OD_600_ =0.5- 0.6) culture was diluted to a 0.5 McFarland standard (OD_600_~ 0.1) in prereduced broth. The diluted culture was inoculated onto duplicate BAKHS agar plate using a cotton tip applicator. The inoculum was allowed to dry before the Metronidazole E-test strip (Metronidazole MZH 256-S30, bioMérieux Australia) was placed in duplicate on the surface of the agar. The agar plate was inverted and incubated for up to 96 hr. The MIC was read as the point at which the elliptical zone of inhibition intercepted the E-test strip at 24, 48 and 96 hr.

### Determination of Minimum Inhibition Concentration (MIC) against *H. pylori*

*Helicobacter pylori* strains, with a range of resistance levels to metronidazole (256 µg/ml, 64 µg/ml, 24 µg/ml, 2 µg/ml, and 0.04 µg/ml), were selected and obtained from Helicobacter Research Laboratory, University of Western Australia. The resistance strength to metronidazole was recorded according to eTest (bioMérieux). *H. pylori* strain 26695 was used as the control. *H. pylori* was maintained on the non-selective Columbia blood agar plates (CBA) (Columbia agar base Oxoid) with 5% horse blood and incubated at 37 °C and 10% CO_2_ for 48 hrs. The MIC of each strain for each compound was obtained using the CLSI agar dilution method, with final concentration of compound 0.0125 - 64 µg/ml.^9^ A control plate that contain 3% DMSO and one without any antibiotics were included in this study. Each of the *H. pylori* strains were harvested, OD_600_ calibrated to 0.3, and spotted in triplicate on CBA pre-mixed with the compound. The lowest concentration of antibiotic showing no growth was read as the MIC. The experiment was performed in duplicate.

### Maintenance of *E. histolytica* and *G. lamblia*

Trophozoites of *E. histolytica* strain HM1:IMSS and *G. lamblia* strains WB, BRIS/87/HEPU/713 (713),^10^ BRIS/83/HEPU/106 (106)^11^ and the metronidazole resistant syngenic line 713-M3^10,11^ were axenically maintained in TYI-S-33 medium supplemented with penicillin (100 U/ml), streptomycin (100 µg/ml).^12^ *E. histolytica* and *G. lamblia* trophozoites were counted using a particle counter (Beckman Coulter, Fullerton, CA). All experiments were performed using trophozoites harvested during the logarithmic phase of growth.

### Determination of EC_50_ against *E. histolytica* and *G. lamblia* trophozoites

The compounds were screened for activity against *E. histolytica* and *G. lamblia* using an ATP-bioluminescence based assay for cell growth and survival.^13,14^ Briefly, 2.5 µL of 5 mM stock compounds were diluted with 17.5 µL sterile water to yield 625 µM working concentration of compounds. A three-fold serial dilution was then performed yielding a concentration range 0.25-625 µM. From this dilution plate, 4 µL were transferred into the 96-well screen plates followed by addition of 96 µL of trophozoites (5,000 parasites) to yield a final 8-point concentration range spanning 0.01- 25 µM. Assay plates were incubated for 48 h at 37 °C in the GasPak^TM^ EZ Anaerobe Gas Generating Pouch Systems (VWR, West Chester, PA) to maintain anaerobic condition throughout the incubation period. The assays were performed in triplicate using the CellTiter-Glo Luminescent Cell Viability Assay.^13^

### Cytotoxicity

HEK293 and HepG2 cells were seeded as 3000 and 5000 cells per well in 384-well plates, respectively. Cells were cultured in DMEM with 1% FBS for 24 hours at 37 °C, 5% CO_2_. Then a dilution series of compounds was added into each well, with the highest concentration of 100 µM. The final concentration of DMSO in culture media was adjusted to 0.5%, which showed no effect on cell growth. After 24 hours incubation with the compounds, 5 µM resazurin were added into each well and incubated at 37 °C for 2 hours. As a negative control, 1% Triton X-100 was added into the culture media to lyse all the cells. The fluorescence intensity was read using Polarstar Omega with excitation/emission 560/590. The data was analysed by GraphPad Prism software. Results are presented as the average percentage of control ± SD using the following equation: Percentage of Growth = (FI_TEST_ – FI_Negative_/FI_UNTREATED_ –FI_Negative_)*100.

### Correlation analysis of compound activity and properties

A correlation matrix between compound activity and properties was calculated using Excel correlation analysis (Table S4). The compound properties, logP, MW, logD, logS and tPSA, were calculated from the 2D structure of the compounds, using Pipeline Pilot (Accelrys, Version 8.5.0.200). The activity was expressed as -log_10_ values of MIC or EC_50_, using average MIC (mol L^-1^) of MtzS *C. difficile* ATCC BAA-1382, ATCC 43255, M7404, ATCC BAA-1803 strains and EC_50_ (mol L^-1^) against *G. lamblia* WB strain and *E. histolytica* HM1:1MSS strain. For correlation between logD and individual activities the linear regression analysis in Excel was used, extracting the linear regression coefficients (R^2^) (Figure S1).

## Supplementary Results: Tables S1-3, Figure S1

### MIC results of *Clostridium difficile* at 24 and 48 hr

Table S1: *Clostridium difficile* MIC values measured after 24 hr and 48 hr incubation for the metronidazole sensitive control strain NAP1/027 ATCC BAA-1803, the metronidazole resistant CD26A54_R strain and the parent sensitive CD26A54_S strain with intermediary resistance to metronidazole.

| Compound | | MIC (µg/mL) | | | | | |
| --- | --- | --- | --- | --- | --- | --- | --- |
|  |  | *C. difficile* NAP1/027 | | | | | |
|  |  | ATCC BAA-1803 | | CD26A54_S | | CD26A54_R | |
|  | | 24 hr | 48 hr | 24 hr | 48 hr | 24 hr | 48 hr |
| **1** | metronidazole | 0.5 | 0.5 | 1 | 1 | 2 | 4 |
| **3** | azide | 0.5 | 1 | 2 | 2 | 4 | 8 |
| **4a** | phenyl | 0.5 | 0.5 | 1 | 1 | 2 | 2 |
| **4b** | p-OMe phenyl | 0.5 | 0.5 | 1 | 1 | 4 | 4 |
| **4c** | p-Cl phenyl | 0.25 | 0.5 | 1-2 | 2 | 8 | 16 |
| **4d** | p,m-Cl_2_phenyl | 1 | 0.5-8 | 1 | 1 | 8-16 | 8-16 |
| **4e** | p-Me phenyl | 0.5 | 0.5 | 1 | 1 | 2 | 2 |
| **4f** | methyl ester | 1 | 1 | 1 | 2 | 4 | 4 |
| **4g** | pyrazole | 1 | 1-2 | 2 | 2 | 4 | 4 |
| **4h** | pyridine | 0.5 | 0.5-1 | 1 | 1 | 2 | 4 |
| **4i** | 5-pyrimidine | 1 | 1 | 1 | 1 | 2 | 4 |
| **4j** | 2-pyrimidine | 2 | 2 | 2 | 2-4 | 4 | 4 |
| **4k** | benzyl | 0.5 | 0.5 | 1 | 1 | 2 | 4 |
| **4l** | CHOH-phenyl | 0.5-1 | 1 | 2 | 2 | 4 | 4 |
| **4m** | CH_2_NMe-benzyl | 2 | 1-2 | 2 | 2 | 8 | 8 |
| **4n** | thiophene | 0.5 | 0.5 | 1 | 1 | 2 | 2 |
| **4o** | thiomorpholine | >64 | >64 | >64 | >64 | >64 | >64 |
| **4p** | CH_2_CH_2_OH | 8 | 8-16 | 8 | 8 | 8-16 | 16 |
| **4q** | CH_2_OH | 16 | 16-32 | 16 | 16-32 | 16 | 16 |
| **4r** | CH_2_CH_2_COOH | >64 | >64 | >64 | >64 | >64 | >64 |
| **4s** | COOH | 64 | 64 | >64 | >64 | >64 | >64 |
| **4t** | CH_2_NH_2_ | >64 | >64 | >64 | >64 | >64 | >64 |

### Metronidazole E-test

Table S2: Metronidazole E-test MIC (µg/mL) determined on BAKHS.

| Time (hrs) | Metronidazole E-test MIC (µg/mL) | | |
| --- | --- | --- | --- |
|  | *C. difficile* NAP1/027 | | |
|  | ATCC BAA-1803 | CD26A54_S | CD26A54_R |
| 24 | 2 | 6 | 32 |
| 48 | 2 | 8 | 48 |
| 96 | 2 | 8 | 48 |

### Comparison of purified and crude Mtz-triazole activity against *G. lamblia* strains

Table S3: Activity (EC_50_) of selected purified Mtz-triazoles (>95% purify) against *G. lamblia* strains compared to literature values^15^ for crude Mtz-triazoles (>85% purity).

| Compound | | EC_50_ (µM) | | | | | |
| --- | --- | --- | --- | --- | --- | --- | --- |
|  |  | *G. lamblia* | | | | | |
|  |  | 106 | | 713 | | 713M | |
|  |  | MtzS | | MtzS | | MtzR | |
|  | | This study | Lit.^15^ | This study | Lit.^15^ | This study | Lit.^15^ |
| **1** | metronidazole | 2.8 | 3.6 | 2.3 | 2.0 | 17 | 50 |
| **4a** | phenyl | 0.28 | 0.26 | 0.16 | 0.16 | 2.3 | 5.3 |
| **4d** | p,m-Cl_2_-phenyl | 1.1 | 1.4 | 2.5 | 0.31 | 1.2 | 0.57 |
| **4e** | p-Me-phenyl | 0.29 | 2.1 | 0.29 | 0.53 | 1.0 | 8.1 |
| **4h** | pyridine | 0.38 | 0.56 | 0.28 | 0.35 | 0.95 | 14 |
| **4k** | benzyl | 0.51 | 0.71 | 0.32 | 0.39 | 1.1 | 5.2 |
| **4m** | CH_2_NMe-benzyl | 1.1 | 0.30 | 0.71 | 0.91 | 2.2 | 9.3 |
| **4n** | thiophene | 0.34 | 0.34 | 0.18 | 0.23 | 0.9 | 6.9 |

### Correlation matrix of compound activity and properties

Table S4: Correlation analysis of compound activity and properties.

|  | *C. difficile* | *E. histolytica* | *G. lamblia* | *logP* | *MW* | *logD* | *logS* | *tPSA* |
| --- | --- | --- | --- | --- | --- | --- | --- | --- |
| *C. difficile* | 1.00 |  |  |  |  |  |  |  |
| *E. histolytica* | 0.68 | 1.00 |  |  |  |  |  |  |
| *G. lamblia* | 0.86 | 0.77 | 1.00 |  |  |  |  |  |
| logP | 0.70 | 0.72 | 0.89 | 1.00 |  |  |  |  |
| MW | 0.14 | 0.01 | 0.36 | 0.56 | 1.00 |  |  |  |
| logD | 0.82 | 0.69 | 0.91 | 0.92 | 0.47 | 1.00 |  |  |
| logS | -0.58 | -0.46 | -0.78 | -0.91 | -0.77 | -0.83 | 1.00 |  |
| tPSA | -0.70 | -0.71 | -0.63 | -0.61 | 0.04 | -0.64 | 0.46 | 1.00 |


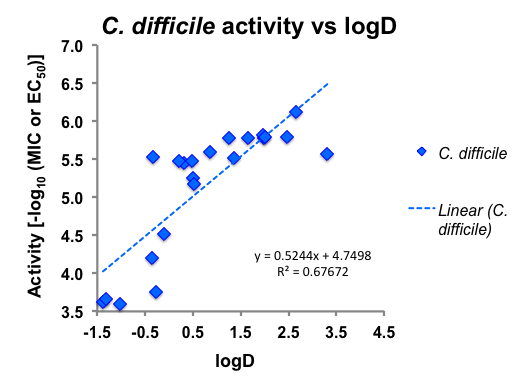

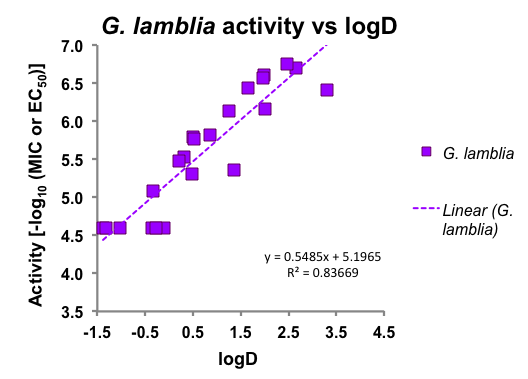

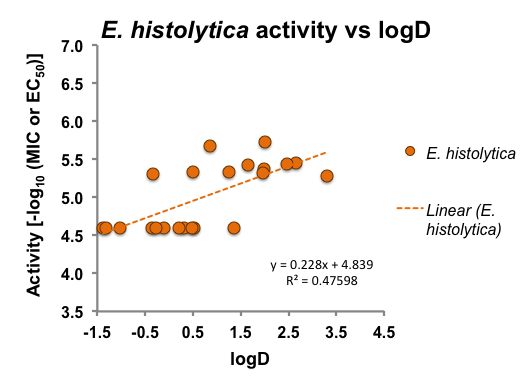


Figure S1: Activity vs logD for individual organisms with linear regression analysis.

## References

(1) Dreier, L., and Wider, G. (2006) Concentration measurements by PULCON using X-filtered or 2D NMR spectra. *Magn Reson Chem* *44 Spec No*, S206–12.

(2) Díez-Barra, E., La Hoz, A. D., Sánchez-Migallón, A., and Tejeda, J. (1990) Synthesis of N-alkylpyrazoles by phase transfer catalysis without solvent. *Synthetic Communications* *20*, 2849–2853.

(3) Mohr, F., Mendía, A., and Laguna, M. (2007) Platinum (II) alkynyl complexes containing N‐and S‐propargylated ligands: synthesis, structures and formation of PtII/AgI coordination compounds. *Eur. J. Inorg. Chem.* 3115–3123.

(4) CLSI. (2012) Methods for dilution antimicrobial susceptibility tests for bacteria that grow aerobically; Approved Standard—Ninth Edition (Wayne, P. A., Ed.), pp 1–88.

(5) Sorg, J. A., and Dineen, S. S. (2009) Laboratory maintenance of *Clostridium difficile*. *Curr. Protoc. Microbiol.* *Chapter 9*, Unit 9A.1.

(6) Lynch, T., Chong, P., Zhang, J., Hizon, R., Du, T., Graham, M. R., Beniac, D. R., Booth, T. F., Kibsey, P., Miller, M., Gravel, D., Mulvey, M. R., Canadian Nosocomial Infectious Surveillance Program (CNISP). (2013) Characterization of a stable, metronidazole-resistant *Clostridium difficile* clinical isolate. *PLoS One* *8*, e53757.

(7) Babakhani, F., Seddon, J., Robert, N., Shue, Y.-K., and Sears, P. (2010) Effects of inoculum, pH, and cations on the *in vitro* activity of fidaxomicin (OPT-80, PAR-101) against *Clostridium difficile*. *Antimicrob. Agents Chemother.* *54*, 2674–2676.

(8) Babakhani, F., Bouillaut, L., Gomez, A., Sears, P., Nguyen, L., and Sonenshein, A. L. (2012) Fidaxomicin inhibits spore production in *Clostridium difficile*. *Clinical Infectious Diseases* *55 Suppl 2*, S162–9.

(9) (2013) Methods for antimicrobial dilution and disk susceptibility testing of infrequently isolated or fastidious bacteria; approved guideline- second addition. *Clinical and laboratory standards institute* 45 ed. Clinical and Laboratory Standards Institute, Pennsylvania.

(10) Townson, S. M., Laqua, H., Upcroft, P., Boreham, P. F., and Upcroft, J. A. (1992) Induction of metronidazole and furazolidone resistance in *Giardia*. *Trans. R. Soc. Trop. Med. Hyg.* *86*, 521–522.

(11) Boreham, P. F. L., Phillips, R. E., and Shepherd, R. W. (1988) Altered uptake of metronidazole in vitro by stocks of *Giardia intestinalis* with different drug sensitivities. *Trans. R. Soc. Trop. Med. Hyg.* *82*, 104–106.

(12) Diamond, L. S., Harlow, D. R., and Cunnick, C. C. (1978) A new medium for the axenic cultivation of *Entamoeba histolytica* and other *Entamoeba*. *Trans. R. Soc. Trop. Med. Hyg.* *72*, 431–432.

(13) Debnath, A., Parsonage, D., Andrade, R. M., He, C., Cobo, E. R., Hirata, K., Chen, S., García-Rivera, G., Orozco, E., Martínez, M. B., Gunatilleke, S. S., Barrios, A. M., Arkin, M. R., Poole, L. B., McKerrow, J. H., and Reed, S. L. (2012) A high-throughput drug screen for *Entamoeba histolytica* identifies a new lead and target. *Nature Medicine* *18*, 956–960.

(14) Tejman-Yarden, N., Miyamoto, Y., Leitsch, D., Santini, J., Debnath, A., Gut, J., McKerrow, J. H., Reed, S. L., and Eckmann, L. (2013) A reprofiled drug, auranofin, is effective against metronidazole-resistant *Giardia lamblia*. *Antimicrob. Agents Chemother.* *57*, 2029–2035.

(15) Miyamoto, Y., Kalisiak, J., Korthals, K., Lauwaet, T., Cheung, D. Y., Lozano, R., Cobo, E. R., Upcroft, P., Upcroft, J. A., Berg, D. E., Gillin, F. D., Fokin, V. V., Sharpless, K. B., and Eckmann, L. (2013) Expanded therapeutic potential in activity space of next-generation 5-nitroimidazole antimicrobials with broad structural diversity. *Proc. Natl. Acad. Sci. U. S. A.* *110*, 17564–17569.
